# Deciphering Supramolecular Structures with Protein-Protein Interaction Network Modeling

**DOI:** 10.1038/srep16341

**Published:** 2015-11-09

**Authors:** Toshiyuki Tsuji, Takao Yoda, Tsuyoshi Shirai

**Affiliations:** 1 Nagahama Institute of Bio-Science and Technology, and Japan Science and Technology Agency, Bioinformatics Research Division, Nagahama, Shiga 526-0829, Japan

## Abstract

Many biological molecules are assembled into supramolecules that are essential to perform complicated functions in the cell. However, experimental information about the structures of supramolecules is not sufficient at this point. We developed a method of predicting and modeling the structures of supramolecules in a biological network by combining structural data of the Protein Data Bank (PDB) and interaction data in IntAct databases. Templates for binary complexes in IntAct were extracted from PDB. Modeling was attempted by assembling binary complexes with superposed shared subunits. A total of 3,197 models were constructed, and 1,306 (41% of the total) contained at least one subunit absent from experimental structures. The models also suggested 970 (25% of the total) experimentally undetected subunit interfaces, and 41 human disease-related amino acid variants were mapped onto these model-suggested interfaces. The models demonstrated that protein-protein interaction network modeling is useful to fill the information gap between biological networks and structures.

Many biological molecules such as proteins and nucleic acids are assembled into non-covalently assembled complexes (supramolecules) to perform complicated functions in the cell. The complex formation is either stable or temporary depending on the nature of the molecular interactions among subunits. The composition, duration, and structure of complexes are very relevant to the specific biological functions of the complexes^1^,^2^. Therefore, a study of protein-protein interaction (PPI) networks of biological molecules in atomic detail is essential for understanding biological activity. However, experimental determination of supramolecular structures is still considerably challenging, especially when a complex consists of hetero-subunits, and computer-aided molecular modeling methods are required to extend structural studies of PPI networks^3^,^4^.

Many previous studies have focused on elucidating the nature of PPI networks by using protein three-dimensional (3D) structures. Kim *et al.* reported one of the early attempts at correlating protein 3D structures to PPI^5^. They suggested that the proteins in the interaction networks could be categorized into two classes. The proteins in one category, namely those in the multi-interface hub, interact simultaneously with multiple partner proteins. On the other hand, proteins in the singlish-interface hub (SIH) category contained proteins with a single interface, which temporarily interact with various partner proteins one at a time. In subsequent comprehensive analyses, the role of intrinsically disordered proteins at hubs was examined, and it was proposed that proteins at the SIH had a tendency to be disordered^6^,^7^.

In other approaches, the protein 3D structures were used for predicting and annotating PPI networks^8^,^9^. Those studies dealt mainly with the binary interactions of proteins in the PPI network. The recently developed database of comprehensive interactome prediction, Interactome3D^10^, comprehensively tabulated as many as 12,167 binary interactions, which are modelable with the experimental structures in the Protein Data Bank (PDB)^11^. Mosca *et al.* developed a pipeline for building dimeric structural models of PPI networks by combining homology modeling and structure-based domain-domain docking methods^12^. With their method, 7% of the experimentally detected binary interactions could be modeled.

However, many biologically interesting protein complexes consist of more than two subunits, and the binary interaction models should be compiled into multi-subunit structures in the PPI network by using appropriate computational methods. In this study, a method of modeling multi-subunit complex models, which will be called PPI complex models, was devised by combining information from PDB and IntAct databases^13^. This method is based on a simple iteration of superposition of binary (dimeric) complexes that share common subunits.

## Results

### Constructing a PPI structure element dataset

The elements of the structures of supramolecules in the PPI network are binary complexes of proteins. Therefore, known structures of interacting binary complexes were sought by combining the structure and interaction databases first. As of May 2014, the interaction database IntAct contained a total of 205,685 binary interactions. The sequences of the interacting proteins were compared with that of the structure database PDB (as of June 2014) to find structures in which homologues of the interacting proteins were determined in complex, which would be called the PPI structure element. Homologous proteins from other species were used for modeling, for example, human complexes when they showed significant sequence identity.

The binary interactions in the IntAct database were classified into four categories. For the complex category, homologous proteins of an interacting pair were, at least partially, determined in complex, and therefore the structures might serve as modeling templates. For the independent category, the homologous protein structures of both sides of the interaction were determined, but they and their homologues were not determined in complex. The interactions in one-sided or unknown categories have only one or none of the structures of interacting proteins, respectively.

For the complex category, the interface in the corresponding binary complex should be part of the total interface between proteins, even if the protein structures have been determined only for part of a whole protein. On the other hand, for the independent category, the partially determined structures might not be involved in the interactions. Therefore, except for those in the complex category, interacting proteins were not further considered in the present study.

**Figure 1:**
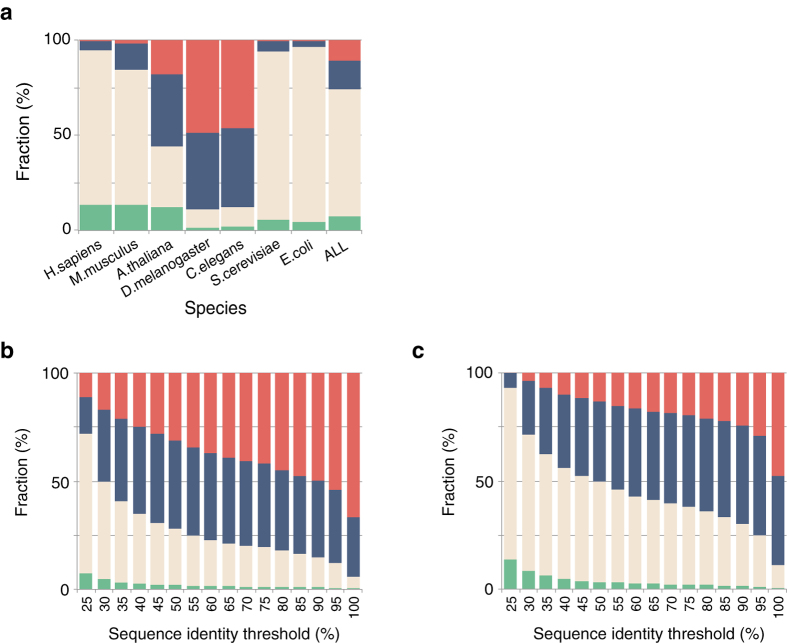
PPI structure elements. (**a**) Binary interactions were fractionated into four categories as complex (homologous complex structure experimentally determined; green), independent (homologous subunit structures of interacting pair determined independently; brown), one-sided (homologue structure of one of interacting pair determined; blue), and unknown (none of the structures of interacting pair determined; red) for different model organisms and all organisms. (**b**) Category fractions against the threshold of sequence identity for PPI structure element assignment for all model organisms and (**c**) for humans (see Figure S1 for other organisms).

In a PPI structure element search, only 8% of total interactions in the IntAct database for all organisms were found to be in the complex category (Fig. 1a). Among those remaining, the independent, one-sided, and unknown categories, contained 67%, 15%, and 10% of the interactions, respectively. When the interactions were separately classified for each organism, the category proportions were found to be largely different. The complex or independent categories accounted for more than 80% of human, mouse, yeast, and bacteria (*Escherichia coli*) interactions. However, the corresponding values for insect (*Drosophila melanogaster*), worm (*Caenorhabditis elegans*), and plant (*Arabidopsis thaliana*) interactions were rather small.

Figure 1b,c, and S1 show the relationship between the sequence identity threshold (25–100%) and category proportions. The number of available PPI structure elements with a sequence identity threshold higher than 40% is less than half of that with a 25% identity threshold. Aloy *et al.* reported the relationship between sequence identity and interaction root mean square deviation (interaction RMSD), which was the RSMD between 14 points (fixed at the center of the mass of each subunit) after superposing A-A′ and B-B′ when comparing homologous A-B and A′-B′ complexes^14^. They suggested that more than 30–40% sequence identity was required for reliable prediction of binary complex structures. Negroni *et al.* proposed a method of template-based docking for low sequence identity pairs of proteins. They reported that proteins sharing more than 30% identity served as suitable templates in docking prediction. The reliability largely declined within 25–30% identities, and a TMscore of more than 0.6 in superposition was recommended for lower identity templates^15^. Korkin *et al.* demonstrated that the positions of protein-binding sites were conserved among 72% of Structural Classification of Proteins (SCOP) domain families^16^. The average minimum sequence identity among the proteins in the same SCOP family was shown to be ~22%. Considering the previous results, 25% sequence identity was employed as the threshold in assigning PPI structure elements in order to retain as many elements as possible, and the PPI structure elements were further examined in the PPI complex modeling process.

### Modeling PPI complexes

**Figure 2:**
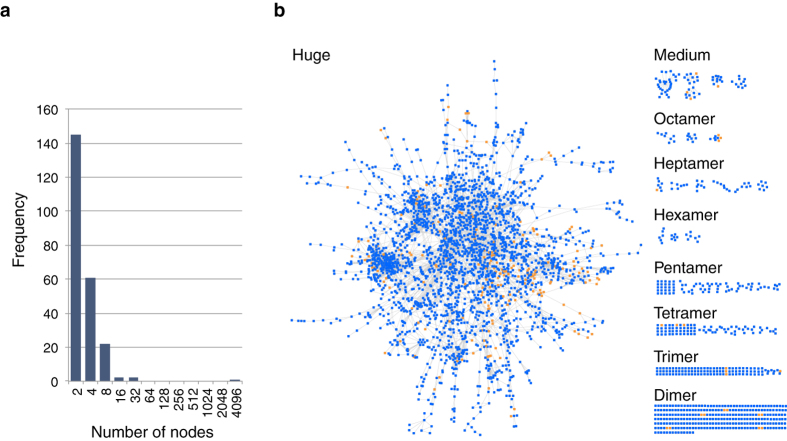
Modelable human PPI sub-networks. (**a**) Size distribution of modelable human PPI sub-networks. The horizontal axis shows the size (number of subunits or nodes in each modelable PPI sub-network. See Figure S2a for definition), and the vertical axis shows the number of sub-networks (see Figure S3 for other organisms). (**b**) Graphs of modelable human PPI sub-networks. Nodes representing interacting proteins are colored blue (used in PPI complex models) or orange (not used). Edges show interaction between proteins.

In order to detect modelable PPI networks, the interactions in the complex category were clustered separately for each model organism (human, mouse, plant, insect, worm, yeast, and bacteria) (Fig. 2a and S3). From the entire graph of the human PPI network, 2,884 nodes (3,436 proteins/domains) and 5,455 edges (interactions) were extracted. They were clustered into 232 PPI sub-networks through single-linkage clustering (Fig. 2b). The resulting sub-networks were called modelable PPI sub-networks, which could be potentially rendered into complex models by referring to the PPI structure element dataset.

**Figure 3:**
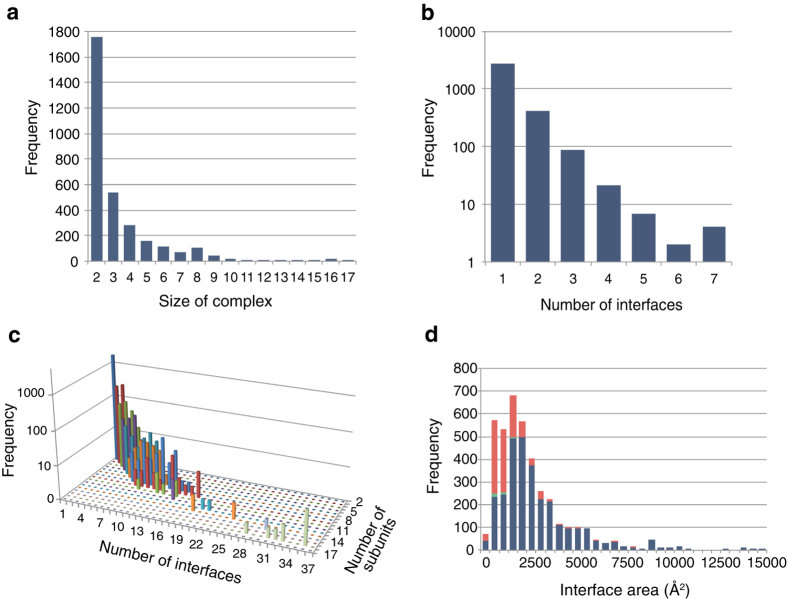
Size and interface distribution of PPI complex models. (**a**) Size distribution of PPI complex models. The horizontal axis shows the size (number of subunits in a model), and the vertical axis shows the model frequency. (**b**) Distribution of number of interfaces on a subunit in PPI complexes (see Figure S2b for definition). The horizontal axis shows the number of interfaces, and the vertical axis shows the logarithmic number of subunits having a corresponding number of interfaces. (**c**) Relationship between numbers of subunits and interfaces in PPI complex models. (**d**) Distribution of interface areas of experimentally determined (in PPI element structures; blue), model-predicted (green), and model-suggested (red) interfaces.

The distribution of the cluster sizes for human PPI sub-networks is shown in Figure 3a. The majority of the sub-networks represented a complex with fewer than nine proteins. Many of the small sub-networks appearing to represent the complex took part in a particular biological function and were readily annotated functionally (Table S1). Notably, medium-size sub-networks consisting of tens to hundreds of subunits were quite rare, although a huge sub-network consisting of 2,240 proteins was detected. This huge sub-network comprised a large variety of functions and cellular localizations from transcription factor to membrane protein, and therefore a specific biological function was difficult to assign.

PPI complex models were constructed by assembling PPI structure elements by superposing common subunits between elements accordingly to the PPI sub-network graphs, and a total of 3,197 models were obtained for humans (Fig. 3a; see Figure S4 for other organisms). The average size of complexes (number of subunits in a complex) was 3.33, and a majority of the complexes, 55% (1,756/3,197), were dimers. The largest complex, namely a 26S proteasome complex, consisted of seventeen homologous subunits.

The protein subunits were assembled by selecting the subunit of the highest degree (number of interacting proteins) in the superposition step. In order to verify this strategy, the model construction was reiterated by selecting anchoring subunits randomly instead of referring to their degree of connection. As the result, distributions of complex size showed no significant difference between the highest degree (3.33 on average) and random selection (3.31) strategies for relatively small complexes. However, the model with the largest number of subunits, the 26S proteasome complex, was constructed only with the highest degree selection strategy, suggesting that this strategy had a slight advantage over random selection. It might be because a subunit with a higher degree allowed a larger number of superposition trials in the construction process.

Theoretically, the modeling procedure employed in this study might produce fibrous protein complex models like actins or microtubules. However, such complexes were not observed among the models obtained. This should be because interfaces that could assemble subunits into infinite periodical fibers were rare in the crystal structures. For example, actin (PDB code 1c0f) and tubulin (4u3j in the PDB) were engineered so that fiber formation was inhibited to obtain crystals^17^,^18^,^19^,^20^.

About 41% (1,306/3,197) of the constructed PPI complex models contained at least one subunit that was absent from the experimentally determined complexes. Particularly, 91% (1,306/1,441) of the models that consisted of more than three subunits contained at least one extra subunit.

The total number of interactions in the PPI complex models was 12,188, of which 7,241 were used for assembling dimers in the modeling process (i.e., the interfaces that were experimentally determined and presented in PPI structure elements), and therefore regarded as experimentally determined interfaces. The remaining interfaces were yielded by modeling, and 105 of them were registered in the IntAct database, although no PPI structure element was found in the PDB. These interfaces are called model-predicted because their structures were predicted by PPI complex modeling for the first time. The other 2,080 interfaces were registered neither in IntAct nor PDB databases, and therefore are called (PPI complex) model-suggested interfaces because they have not been experimentally detected yet. When redundant homologous interfaces (those between homologous proteins pairs) were ignored, these figures included 2,989 experimentally determined, 31 model-predicted, and 970 model-suggested in a total of 3,959 unique interfaces (Table 1).

Figures 3b and S5 shows the distribution of the number of interfaces in proteins. It was found that 80% (2,745/3,436) of the proteins have a single interface for interacting with other proteins. Though it is not surprising, subunits in larger complexes tended to possess more interfaces (Fig. 3c). The proteins with the largest number of interfaces had seven different interfaces. They were the non-ATPase regulatory subunit 7 of 26S proteasome, MAP kinase kinase kinase 3, and growth factor receptor bound protein 2.

The models also revealed that about 35% (1,370/3,921) of the interfaces were alternatives, which had more than two interacting proteins but could bind only one of them at a time. Among the predicted alternative interfaces, 44% (606/1,370) were used to interact with proteins homologous to each other (i.e., protein A interacted with proteins B or C at a single interface and B and C were homologs of each other), and 24% (330/1,370) was used to interact with proteins that might not be homologous but had the same fold with a TMscore within >0.5 of each other. The remaining 32% (434/1,370) of the alternative interfaces appeared to accept proteins of non-homologous and different folds from each other.

### Evaluating PPI complex models

In order to evaluate the accuracy of PPI complex models, a virtual blind test of the models against experimentally determined structures was carried out. Due to the modeling strategy of the present study, some of the PPI complex models were equivalent to, part of, or contained the corresponding experimentally determined complexes. In some cases, however, the models were constructed by referring to PPI elements from different experimental structures (PDB entries).

For example, a model assembling PPI elements A′-B′ and B′-C′ might be compared with an experimentally determined A-B-C complex, either as a whole or by relative position of (predicted) subunit C (C′) to others. A total of 118 PPI complex models and 145 subunits were found to fall into this category. Figures S6a and S6b show the distribution of the overall rmsd of whole models and the predicted subunits, respectively. The overall rmsd was 1.78 Å on average, and 100% and 84% (122/145) were smaller than 10 and 5 Å, respectively.

The rmsd of predicted subunits was 1.87 Å on average, and 100% and 99% (144/145) were smaller than 10 and 5 Å, respectively. These values were comparable to l-rms values (rmsd of the ligand subunit in protein-protein docking predictions) employed in the critical assessment of predicted interactions (CAPRI)^21^. In CAPRI conventions, these values of 10, 5, and 1 Å represent the upper limits of acceptable, medium, and high accuracies in prediction. According to the convention, 100%, 99% (144/145), and 23% (33/145) of the predicted subunits in the PPI complex models had acceptable, medium, and high accuracies, respectively. The model accuracy did not show significant variation over sequence identity between predicted and template subunits (Figure S6c) or size of complex (Figure S6d).

### Mapping variants of amino acids on PPI complex models

Because interactions in PPI networks are essential for biological activity, a considerable number of human diseases are thought to be caused by impaired PPI complexes^22^,^23^. Therefore, the constructed models were used for mapping human amino acid variants. In order to extrapolate the constructed PPI complex models from those not explicitly detected at this point, homologous proteins were aligned to the corresponding subunits in the PPI complex models. This procedure generated 9,512 complex models with 10,623 interfaces (Table 1).

The amino acid variants defined in the OMIM database are classified into three types, namely, polymorphism, disease, and unclassified, and contain 37,916, 24,608 and 6,562 variants, respectively. They were mapped on the models according to the UniProt definition^24^.

Table 1 shows the statistics of the molecular environments of variant amino acid residues of the extrapolated PPI complex models. Approximately half of the variants were mapped onto undetermined or unstructured regions, and 16% (859/5,254), 57% (2,990/5,254), and 27% (1,405/5,254) of the remaining variants were mapped onto interface, surface, and interior (buried residues) of the PPI complex models, respectively. For disease-related variants, 15% (321/2,095), 48% (1,014/2,095), and 36% (760/2,095) of the variants of the structured regions, were mapped onto the interface, surface, and interior, respectively. Notably, 12% (41/331) of human disease-related variants were mapped onto experimentally undetected but PPI complex model–suggested interfaces (Table S2).

## Discussion

The current database revealed that only 14% (8% of all organisms) of total detected interactions of human proteins could be assigned to 3D structures when binary complexes of proteins called PPI structure elements were sought by combining the structure and interaction databases (complex category in Fig. 1a). Only 0.5% (241/45,189) of them were experimentally determined as themselves, i.e., the experimental complex structure of IntAct registered proteins, and the majority (5,979/6,220) were those modelable from homologous complexes. The human interactome has been thought to contain ~130,000 binary interactions^25^, and ~35% are registered in the IntAct database already. If the observed proportions were simply extrapolated, about 18,000 of total human interactions would be modelable from the current structure database. These results are consistent overall with the previous report^10^.

The low coverage of PPI structure elements implied that the current structure data might be rather insufficient to elucidate the PPI structure as a whole. The detected PPI structure elements were reconstructed into modelable PPI sub-networks, and the majority (55%, 1,756/3,197) of the sub-networks were found to be complexes with two proteins. Many of the small sub-networks could be functionally annotated (Table S1). They typically represented stable (permanent) complexes of membrane proteins (including membrane-bound proteins) and mitochondrial proteins, consisting of 38% (281/734) membrane proteins and 52% (75/143) mitochondrial proteins in sub-networks. The enrichment of these proteins in small sub-networks was significant, with *p*-values of 1.4 × 10^−12^ for membrane proteins and 7.9 × 10^−10^ for mitochondrial proteins by a hypergeometric distribution test. The enrichment might be partly because experimental structure analysis of membrane protein complexes has been difficult. Considering that a mitochondrion is a membrane-compartmented organelle, another explanation would be that a membrane acts as a barrier to a PPI network and the network tends to be disrupted at the membrane.

Notably, however, the largest sub-network detected consisted of 2,240 proteins with a large variety of functions and cellular localizations and therefore was difficult to characterize functionally as a whole. The presence of the huge sub-network implied that a considerable number of complex models could be derived from it. Actually, the modeling attempts generated 3,197 supramolecule models, in which 41% (1,306/3,197) of the models contained at least one subunit that was absent from the experimentally determined complex structure, and 17% (2,080/12,188) of their inter-subunits interfaces were not experimentally detected yet. These model-predicted (interaction known but structure unknown) or -suggested (both interaction and structure unknown) interfaces should be the major products of the modeling, which are difficult to derive by other means.

The majority of the model-predicted or -suggested interfaces tended to have smaller interaction areas in comparison to those (experimentally determined) from known structures (Fig. 3d). This might suggest that considerable numbers of the interfaces in these categories are artifacts of modeling. However, the result from disease-related variant mapping implied they are functionally significant, as discussed in a later section in detail (Table 1). Because these model-derived interfaces are peripheral to the major interactions, they have most likely been missed in interaction-detecting experiments that were oriented to binary interactions, such as yeast-two-hybrid or pull-down methods.

**Figure 4:**
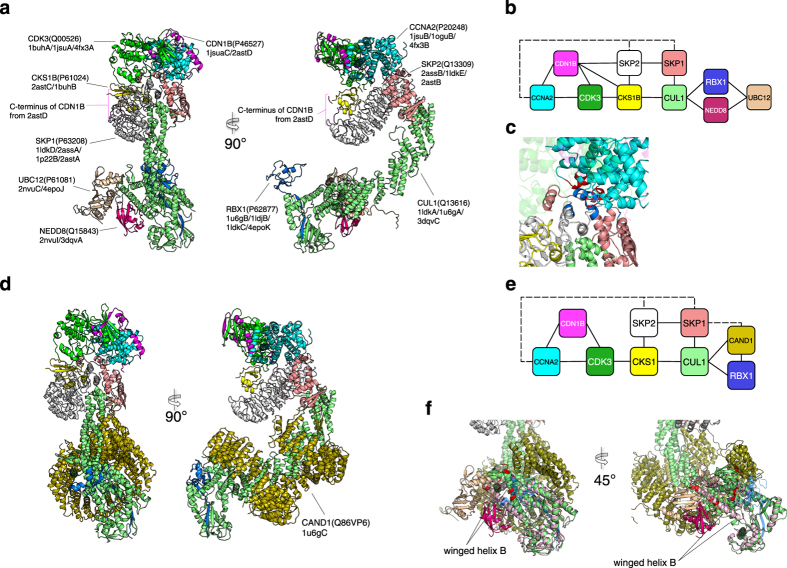
Cyclin-A2 complex model. (**a**) Cyclin-A2 complex model in front and side views. Subunits presented are cyclin A2 (CCNA2; shown in cyan n models), cyclin-dependent kinase 3 (CDK3; green), cyclin-dependent kinase inhibitor 1B or p27Kip1 (CDN1B; magenta), cyclin-dependent kinase regulatory subunit 1 (CKS1; yellow), S-phase kinase-associated protein 1 (SKP1; pink), S-phase kinase-associated protein 2 (SKP2; gray), cullin-1 (CUL1; lime), E3 ubiquitin-protein ligase RBX1 (RBX1; blue), NEDD8 (NEDD8; purplish red), and NEDD8-conjugating enzyme Ubc12 (UBC12; light brown). UniProt IDs, PDB codes, and chain IDs of subunits used for constructing models are also shown for each subunit in parentheses. (**b**) Modelable PPI sub-network for cyclin-A2 complex. The solid black and dotted black lines indicate IntAct-registered and model-suggested interactions, respectively. (**c**) Close-up of the model-suggested interfaces between CCNA2 (cyan) and SKP1 (pink), and CCNA2 and SKP2 (gray). The residues at the interface are colored red for CCNA2 and blue for SKP1 and SKP2. (**d**) Inhibited state cyclin-A2 complex model in front and side views. Two subunits, NEDD8 and UBC12, were removed from the model in Panel a, and cullin-associated NEDD8-dissociated protein 1 (CAND1, olive) was introduced. (**e**) Modelable PPI sub-network of inhibited state cyclin-A2 complex model. The solid black and dotted black lines indicate IntAct-registered and model-suggested interactions, respectively. (**f**) CUL1 C-terminal domains (lime) are superposed between non-inhibited (Panel a) and inhibited (Panel d) complexes. Residues in alternative interface for CAND1 and NEDD8 (purplish red) are shown in red.

Detailed inspections of the models suggested several characteristics of PPI complex models and model-derived interfaces. Figure 4a shows a model containing ten subunits based on the modelable network proximal to cyclin A2. The complex of cyclin A2 (CCNA2)-cyclin dependent kinase (CDK3)-cyclin-dependent kinases regulatory subunit 1 (CKS1B) promotes cell cycle transitions from G_0_ to G_1_ and from G_1_ to S phases^26^,^27^. The kinase activity of CDK3 is inhibited by cyclin-dependent kinase inhibitor 1B (CDKN1B) during the G_0_ phase, and polyubiquitination of CDKN1B by E3 ubiquitin-protein ligase complex (SKP1-CUL1-SKP2-RBX1) is required to activate CDK3-CCNA2-CKS1B. SKP2 has an F-box domain that recognizes the polyubiquitination target, the C-terminus of CDKN1B in this case. SKP1 is the core component of E3 ubiquitin ligase complex, which works as an adaptor between SKP2 and CUL1.

The complex model represents the inhibited CDK3-CCNA2 complex bound to RBX1 with other protein subunits required for this process. RBX1 activity is regulated by ubiquitin-like proteins, such as ubiquitin, SUMO, or NEDD8 attached to Cullin (CUL1). It is thought that the C-terminus domain of CUL1 becomes open on binding a ubiquitin-like protein (NEDD8 in this model). Then, the C-terminus domain of CUL1 leaves CAND1 and RBX1. The untethered RBX1 is essential for E3 ubiquitin ligase activity. Two of the interfaces presented in this model, namely between CCNA2 and SKP1 and between CCNA2 and SKP2, are registered in the IntAct database, but the interacting structure has not been experimentally determined (Fig. 4b). The model demonstrates how CDKN1B would be ubiquitinated. CCNA2-SKP1 and CCNA2-SKP2 would play a role in presenting the C-terminus of CDKN1B, which is known to be polyubiquitinated, to the ubiquitin-protein ligase RBX1^28^.

Hao *et al.* proposed a model of the SKP1-SKP2-CUL1-RBX1-CKS1B-CDKN1B-CDK2-CCNA2 complex by assembling four crystal structures based on a strategy similar to that of the present study^29^. The present study, by comprehensively collecting the PPI structure elements, successfully added two subunits, namely a neural precursor cell expressing developmentally down-regulated protein 8 (NEDD8) and NEDD8-conjugating enzyme Ubc12 (UBC12), to the model (Fig. 4a).

An alternative model of the CDK3-CCNA2-CKS1B regulating complex was generated through the present method (Fig. 4d,e). The model represents a ligation-inhibited state of the complex containing cullin-associated NEDD8-dissociated protein 1 (CAND1). The model shows the open conformation of a NEDD8-modified state, which is essential for ubiquitination activity^30^. If CAND1 is bound to winged-helix B in the C-terminal domain of CUL1, NEDD8 cannot co-exit this complex. In this case, the interface of CUL1 is alternative, and it might explain the inhibition mechanism, in which NEDD8 physically prevents the involvement of CAND1 (Fig. 4f). Thus, the present method can generate alternative complex models in several different states from the PPI network. The PPI complex models suggested that about 35% (1,370/3,921) of the interfaces overall are alternatives like those of CUL1-NEDD8 and CUL1-CAND1.

**Figure 5:**
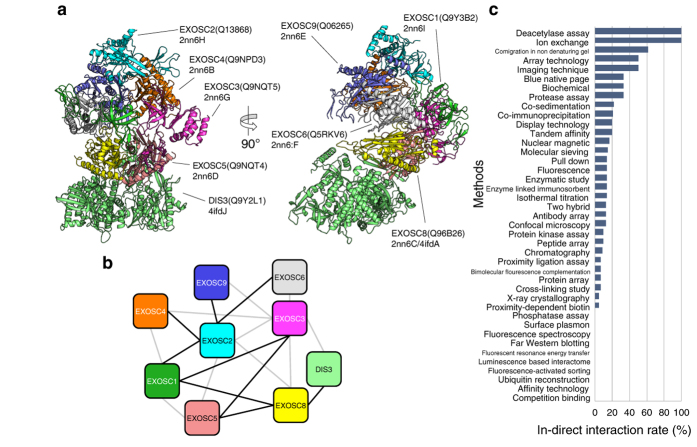
PPI complex model-suggested indirect interactions. (**a**) Front and side views of RNA exosome complex model. Subunits presented are exosome complex components CSL4 (EXOSC1; green), RRP4 (EXOSC2; cyan), RRP40 (EXOSC3; magenta), RRP41 (EXOSC4; orange), RRP46 (EXOSC5; pink), MTR3 (EXOSC6; gray), RRP43 (EXOSC8; yellow), RRP45 (EXOSC9; blue), and exosome complex exonuclease RRP44 (DIS3; lime). (**b**) The modelable PPI sub-network of RNA exosome complex. The black and grey lines indicate direct and indirect interactions, respectively. (**c**) The rate of indirect interactions in total observations in the PPI complex models is shown for each experimental method of molecular interaction analysis. See also Table S2 for the methods for which no indirect interaction was assigned.

As another example, a part of the large complex of the RNA exosome, which is composed of more than nine proteins, was modeled (Fig. 5a). The RNA exosome degrades various RNA molecules in the cell, and the complex consists of nine core proteins (EXOSC1–9) and interacts with a variety of accessory proteins^31^,^32^,^33^. The structures of the RNA exosome complex of humans and yeast have been determined by X-ray crystallography (PDB codes 2nn6 and 4ifd, respectively). Eventually, the PPI complex modeling merged two experimental structures and introduced eight subunits into the human complex or one subunit into the yeast complex.

For the network of a large complex like the RNA exosome, the graphs of PPI networks tend to be complete in the IntAct database; that is, each node (protein) is connected to (interacts with) all the other nodes. In many cases, however, those interactions appear to be indirect in the models. In the RNA exosome model, 14 (out of a total of 23) of the interactions registered in the IntAct database were suggested to be indirect (Fig. 5b).

An inspection of the PPI complex models suggested that 16% (1,480/9,544) of total interactions are indirect. This kind of indirect interaction appeared to be experimentally detected depending on the methods. The predicted indirect interactions tend to be determined by coimmunoprecipitation, cosedimentation, native PAGE, or comigration in non-denaturing gel electrophoresis, which detect the co-existence but physical interaction of proteins (Fig. 5c and Table S3). Figure S7a shows three types of interfaces: experimentally determined (solid line at the bottom of Figure S7b), model-suggested (dotted line), and indirect interfaces (gray line) in the modelable network proximal to actin-related protein 2 (ACTR2, green). Indirect interaction between actin-related protein 3B (ACTR3B, cyan) and actin-related protein 2/3 complex subunit 5-like protein (ARPC5L, blue) was detected via an anti-bait co-immunoprecipitation experiment.

Thus, the present examples of a PPI complex model demonstrate the types of annotation that PPI network modeling might add. Discrimination between alternative and simultaneous interfaces is not made on an interaction network graph in most cases. PPI complex models reveal how many interfaces (at least) a protein has, how many proteins (at least) interact with each interface, and whether the interactions are permanent or not. If an interface is an alternative, interactions on the interface are likely temporal and probably significant for functional regulation. PPI complex models are also useful to discriminate between direct and indirect interactions, which are not readily detected by many of the frequently used experimental methods in proteomics. Annotation of direct/indirect interactions would be important in analyzing the mechanisms of a complex structure, for example, by mapping amino acid variants.

Approximately 52% of the amino acid variants identified for human proteins were mapped on the interior or interface of the PPI complex models. The variants were more enriched on the interface (*p*-value = 4.9 × 10^−11^ by *χ*^2^ test) and interior (*p*-value = 4.3 × 10^−54^) of proteins than expected compared to (benign) polymorphism class variants. This result is consistent with previous studies reporting that detected disease-related mutations are frequently observed in protein interiors or interfaces^23^,^34^,^35^,^36^. Among the 331 disease-related variants mapped to the PPI model interfaces, 12% (41/331) were found on experimentally undetected but PPI complex model–suggested interfaces.

Disease-associated variants per unit area of the model-suggested interface were approximately 0.53 variants per 10,000 Å^2^ interface area, and this value was not significantly different from that for the experimentally determined interfaces. The PPI complex modeling has newly attributed 777,203 Å^2^ (237 Å^2^/subunit on average) out of a total 41,350,629 Å^2^ (12,599 Å^2^/subunit on average) in surface area to possible interfaces with other proteins. This reattribution of 1.9% of the surface area bought 41 (3.9% of 41/1,045) disease-related variants into the interface category from the surface category. The number of reattributed disease-related variants was higher than expected (*p*-value = 7.94 × 10^−4^ by *χ*^2^ test), suggesting a considerable fraction of the disease-related variants for which no relationship to PPI is currently known might be reconsidered by using PPI complex modeling.

**Figure 6:**
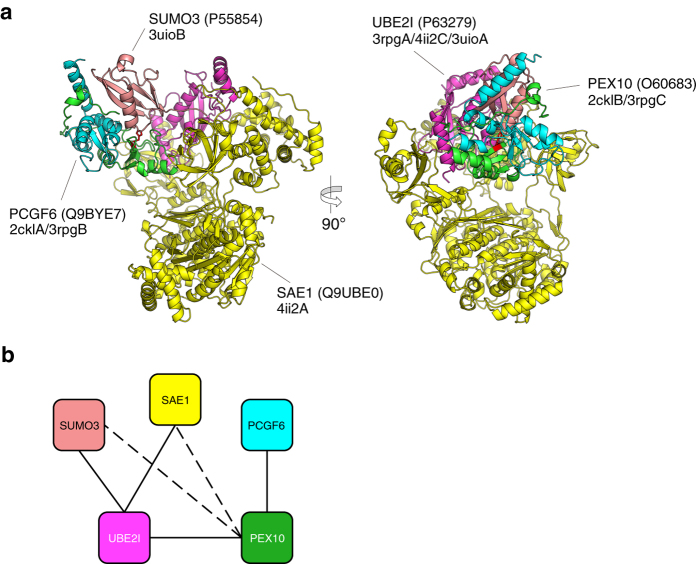
Disease-related variants on PPI complex model-suggested interface. (**a**) Front and side views of a PEX10 complex model containing peroxisome biogenesis factor 10 (PEX10; green), polycomb group RING finger protein 6 (PCGF6; cyan), SUMO-conjugating enzyme UBC9 (UBE2I; magenta), SUMO-activating enzyme subunit 1 (SAE1; yellow), and small ubiquitin-related modifier 3 (SUMO3; pink). The variant site His290 related to peroxisome biogenesis disorder 6B disease is indicated on SUMO3 (stick model in red). (**b**) Modelable PPI sub-network of PEX10. The solid black and dotted black lines indicate direct and model-suggested direct interactions, respectively.

An example of a disease-related variant assigned to a model-suggested interface is presented in Figure 6. The variant of His290 of peroxisome biogenesis factor 10 (PEX10) has been related to peroxisome biogenesis disorder 6B disease^37^. His290 of PEX10 was mapped on the interface to small ubiquitin-related modifier 3 (SUMO3). Because this residue is on the RING-type zinc finger motif, the variant might affect the function of PEX10 itself. However, the model would introduce another explanation that involves disruption of the interaction with SUMO3.

A total of 41 variants associated with 26 kinds of human diseases were mapped to the model-suggested interfaces (Table S2). For 10 diseases, the relevant 13 variants were mapped to a PPI interface for the first time, namely, premature chromatid separation trait, LEOPARD syndrome 1, Charcot-Marie-Tooth disease dominant intermediate type F, multiple self-healing squamous epithelioma, peroxisome biogenesis disorder 6B, lissencephaly 3, precocious puberty central 2, amyotrophic lateral sclerosis 18, thrombocytopenia 1, and leukodystrophy hypomyelinating 6. Thus, the PPI complex models suggest the molecular mechanism of these variants to be a disruption of PPI, and they demonstrate the validity of the method introduced in analyzing disease-related variants.

In summary, comprehensive PPI complex modeling might be employed for annotating PPI network with the natures of the protein interactions, such as alternative/simultaneous or direct/indirect. The PPI complex models also help reveal experimentally undetermined interfaces. The extent of the applicability of this method depends on the number of experimentally determined binary interactions, and it is expected that the method will generate increasingly large numbers of PPI complex structures in the future.

## Methods

### PPI structure element dataset

**Figure 7:**
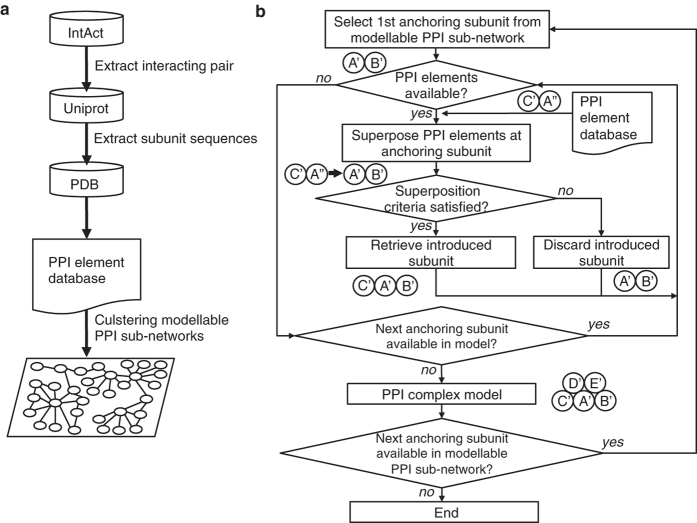
Schemes of data processing and model building procedure. (**a**) Schematic procedure of PPI element database and modelable sub-network preparations. (**b**) Flowchart for PPI complex model construction. Superposition criteria are satisfied if the sequence identity between superposed subunits was more than 30% and TMscore was more than 0.5 or sequence identity was between 25% and 30% and TMscore was more than 0.6.

A dataset of PPI structure elements as building units of the PPI complex model was prepared referring to the PDB and the PPI database, IntAct. PPI structure elements are dimeric protein complexes, for which the homologous subunits are registered in the IntAct database as an interacting protein pair. The amino acid sequences of the proteins in an interacting pair were retrieved from the UniProt database^38^ and were compared with the amino acid sequences of the proteins in the PDB by using BLAST^39^ (Fig. 7a). The sequences in the PDB that showed more than 25% amino acid sequence identity were retrieved for each interacting protein. Sequence identities were calculated for the aligned parts of sequences between template (structure-known homologue) and target (protein for modeling). Then, cocrystallized (or coanalyzed) proteins in the PDB were sought in the retrieved sets, and the proteins with the highest average identity over interacting protein pairs were selected as the templates for further modeling and were called the PPI structure element. Coverage between sequences was not considered at this point of the process.

### Modelable PPI sub-networks

The PPI network of the IntAct database was broken down into sub-networks, for which all of the structures and interactions of interacting pairs were experimentally determined, at least for homologous proteins. The interactions, for which a PPI structure element was assigned, were extracted from the whole network and were applied for single-linkage clustering. Thus, sub-networks were part of an entire PPI network, in which every pair of nodes (proteins) in a sub-network was linked through at least a single path of the edges (protein interaction represented by PPI structure element). The resultant sub-networks were called modelable PPI sub-networks, which could be potentially rendered into complex models by referring to the PPI structure element dataset (Fig. 7a).

### PPI complex modeling

PPI complex modeling was performed according to the detected modelable PPI sub-networks (Fig. 7b). The models were constructed by iteratively compiling the PPI structure elements as follows. Given that proteins A, B, and C are connected in a modelable PPI sub-network, and the PPI structure elements consisting of A′-B′ and A′′-C′ (where A′/A′′, B′, and C′ are homologous to A, B, and C, respectively) were found in the dataset, then a model of the B-A-C complex was built by superposing A′ and A′′. In this example, subunit A′ of the A′-B′ complex was used for the anchoring subunit to assemble subunit C′. Thus, a single superposition introduced one subunit into the complex under construction.

The complex modeling was repeatedly executed by selecting every subunit in the sub-network as the starting point of iteration. Because it often required an unacceptably long computational time to trace all possible paths in a large modelable PPI sub-network, the superposition order was determined by referring to the interaction degree (number of interacting partners in a relevant PPI sub-network). For each modeling attempt, starting subunit A′ served to anchor the subunit, and after each superposition, an anchoring subunit was selected from the subunits in the complex under construction so that the anchoring subunit had the largest interaction degree among the subunits, including those previously used as an anchor. If an anchoring subunit did not introduce any subunit, then the subunit with the second largest degree was selected, and so forth.

Superposition was executed by using TM-align software^40^. If the sequence identity (based on structure alignment) was more than 30% and the TMscore was less than 0.5 or the sequence identity was between 25–30% and the TMscore was less than 0.6, which implied that the anchoring subunit was not adequately superposed (A′′ to A′ superposition in assembling A′-B′ and A′′-C′), the superposition was not accepted and the introduced subunit (C′) was discarded. These superposition criteria were within a reasonable range to assure reliable structure comparisons according to previous studies^15^. The results of superposition were also discarded if the introduced subunit (C′) hindered one of the other subunits (for example, B′) in the model under construction. In this case, the interactions between A-B and between A-C would not be made simultaneously, and they (B-A and C-A for A) were recorded as alternative interfaces.

Because experimental structures were often determined only for interacting domains or sub-domains, it was difficult to judge template qualities solely from amino acid sequence comparison, especially from coverage between target and template. In this study, therefore, PPI elements were retrieved without considering coverage, as mentioned above, and quality control was introduced in the structure superposition steps.

The iterations of superposition were continued until no PPI structure element was available in the database for further superposition, and the resultant models were called PPI complex models. This strategy of using the highest degree selection for the anchoring subunit was compared with that of random selection for verification purposes.

In the current study, comparative (homology) modeling of subunits/complexes according to the sequences of interacting pairs was not executed because the number of models was large. Instead, the sequences of interacting pairs were simply mapped onto that of corresponding PPI structure elements.

The interface between a pair of subunits on a subunit in the models was defined as the buried surface area. An interacting surface with an area of more than 250 Å^2^ was regarded as an interface. Overlapping interfaces were distinguished when they shared less than 10% of areas for different interacting subunits. Surface area was calculated by using DSSP software^41^.

Details of the PDB codes of PPI elements used for modeling are summarized in the supplemental material (Figure S8).

### PPI complex model evaluation

In order to evaluate model accuracy, some of the PPI complex models were compared with experimentally determined structures. Due to the modeling strategy employed, some of the constructed models were equivalent to the corresponding experimentally determined complexes, but they were constructed by referring to PPI elements from different experimental structures (PDB entries). For example, if a complex of subunits A, B, and C was constructed, and the complex structure of A-B-C was experimentally determined but the model was an assemblage of two PDB entries of binary complexes A′-B′ and B′-C′, then the model structure would be used to evaluate modeling accuracy. The PPI complex model A′-B′-C′ served as a virtual blind model of A-B-C in this case, and comparisons of whole complex structures and relative positions of subunit C (C′) to A-B (A′-B′) or A (A′) to B-C (B′-C′) might provide an objective estimation of model accuracy.

By referring to the model construction history, a total of 118 PPI complex models and 145 subunits within those models were found to fall into this category. The selected PPI complex models were either equivalent to, part of, or contained the corresponding experimentally determined complex structure. The complex structures were superposed as a whole to evaluate rmsd. In addition, rmsd values of predicted subunits (A′ or C′ in the above mentioned example) were evaluated by superposing remaining common subunits between complexes.

### Mapping disease-related amino acid variants on PPI complex model

PPI complex models were used for mapping the amino acid variants related to human diseases. For this purpose, the constructed PPI complex models were extrapolated. About 10% (2,094/20,197) of the human proteins for which disease-related amino acid variants were discovered are not registered in the IntAct database. Therefore, the constructed models of human protein complexes were used to analyze possible interactions of those proteins as follows.

The human protein sequences retrieved from the UniProt database were compared with the sequences of the subunits in the constructed PPI complex modes using BLAST. Sequences that showed more than 25% identity to at least one subunit in the models were retrieved, and the models were re-assigned to the alternative homologues. If human proteins A, B, and C interacted and complex model A-B-C was constructed, this model was used to demonstrate possible complexes such as A-B-C′, A-B′-C′, etc… where proteins B′ and C′ are human homologs of proteins B and C, respectively. When a model contained subunits A, B, and C, and the subunits had 2, 3, and 3 homologues, respectively, then a total of 18 trimers were generated according to combinations of the sequences as variations of the A-B-C PPI complex model. If an interface of two human proteins already existed in the original PPI complex model, that is, the interacting pair was registered in the IntAct database, then it was called an experimentally detected interface. Otherwise, interfaces were regarded as model-predicted (if interaction was recorded in IntAct) or -suggested (otherwise) interfaces.

The information on disease-related amino acid variants was retrieved from the OMIM database^42^. The correspondence between amino acid variants in OMIM and amino acid sequences in UniProt was determined according to UniProt descriptions^23^. The amino acid variants were mapped onto the corresponding PPI complex model(s) according to the above-mentioned comprehensive sequence comparison, and the distributions of the mapped variants to different molecular environments were analyzed by hypergeometric distribution analysis^43^,^44^. The molecular environments of the residues were evaluated with DSSP software^41^. A residue with less than 0.1 accessibility on an isolated subunit was thought to be buried. Exposed residues were differentiated into surface or interface (experimental or model-suggested depending on the origin of the interacting subunit) as described above.

## Additional Information

**How to cite this article**: Tsuji, T. *et al.* Deciphering Supramolecular Structures with Protein-Protein Interaction Network Modeling. *Sci. Rep.* **5**, 16341; doi: 10.1038/srep16341 (2015).

## Supplementary Material

Supplementary Information

## Figures and Tables

**Table 1:** Interface types and distribution of amino acid variants over molecular environments in PPI complex models*

Type	Interface			Surface	Interior	Disordered/ no structure	Total
	Experimentally determined	Model-predicted	Model-suggested				
Interface processing							
No. in PPI structure elements	10,003	105	2,080	−	−	−	12,188
No. non-redundant	2,958	31	970	−	−	−	3,959
No. extrapolated	8,633	88	1,902	−	−	−	10,623
Variant assignment							
Polymorphism	261 (155, 1.1 × 10^−17^)	6 (1, 7.5 × 10^−5^)	31 (9, 4.2 × 10^−14^)	1,262 (1,245, 0.62)	352 (557, 3.5 × 10^−18^)	3,106 (3,052, 0.32)	5,018
Disease-related	287 (113, 5.6 × 10^−60^)	3 (1, 0.046)	41 (6, 9.5 × 10^−43^)	1,014(909, 7.0 × 10^−4^)	760 (407, 6.8 × 10^−68^)	1,571 (2,229, 7.2 × 10^−45^)	3,676
Unclassified	218 (65, 2.5 × 10^–79^)	0 (1, 0.45)	22 (4, 1.5 × 10^−21^)	714 (527, 3.8 × 10^−16^)	293 (236, 2.0 × 10^−4^)	878 (1,292, 9.9 × 10^−31^)	2,125
Total	766	9	94	2,990	1,405	5,555	10,819

^*^Expected values under non-biased distribution and *p*-values of observed number are in parentheses.
