# Supplementary material for: Deciphering Supramolecular Structures with Protein-Protein Interaction Network Modeling
Source: Sci Rep. 2015 Nov 9;5:16341. doi: 10.1038/srep16341 (PMC4637837; doi:10.1038/srep16341)
Supplement: Supplementary Information [file srep16341-s1.pdf]

# SUPPLEMENTAL INFORMATION

## Deciphering Supramolecular Structures with Protein-Protein Interaction Network Modeling

**Toshiyuki Tsuji, Takao Yoda, Tsuyoshi Shirai**

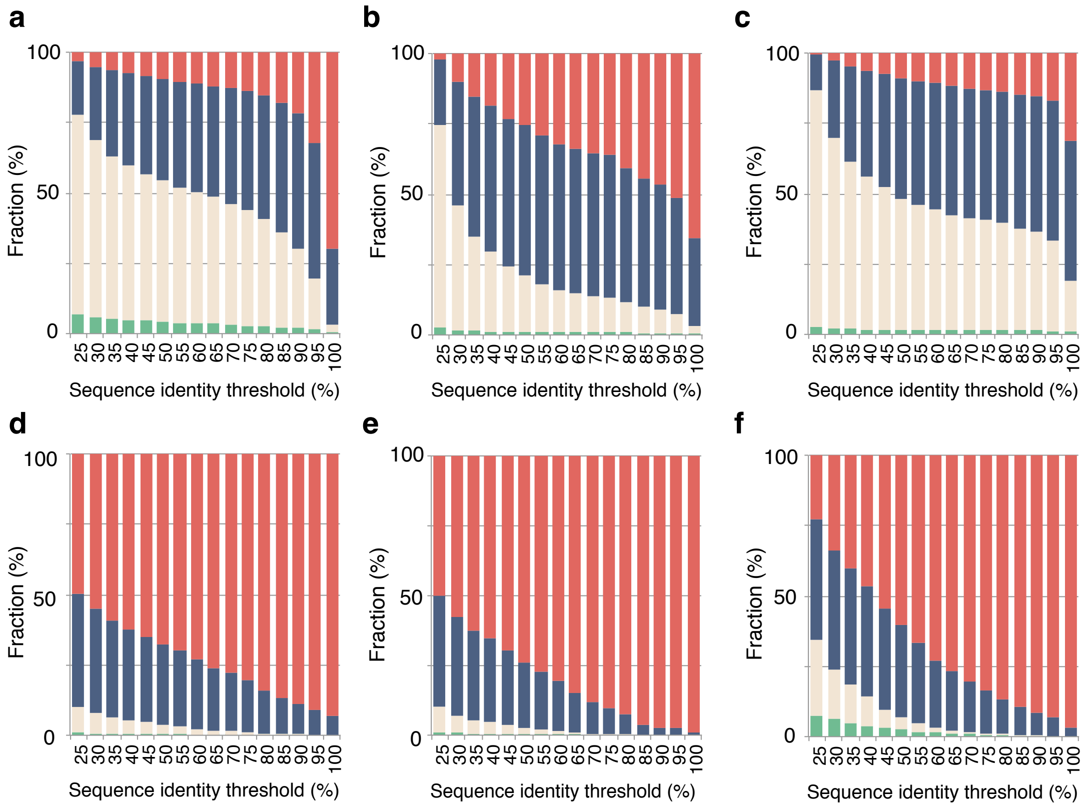


**Figure S1. PPI structure elements**

Category fractions against the threshold of sequence identity in PPI structure element assignments for (a) mouse, (b) yeast, (c) bacteria (*Escherichia coli*), (d) insect (*Drosophila melanogaster*), (e) worm (*Caenorhabditis elegans*), and (f) plant (*Arabidopsis thaliana*).

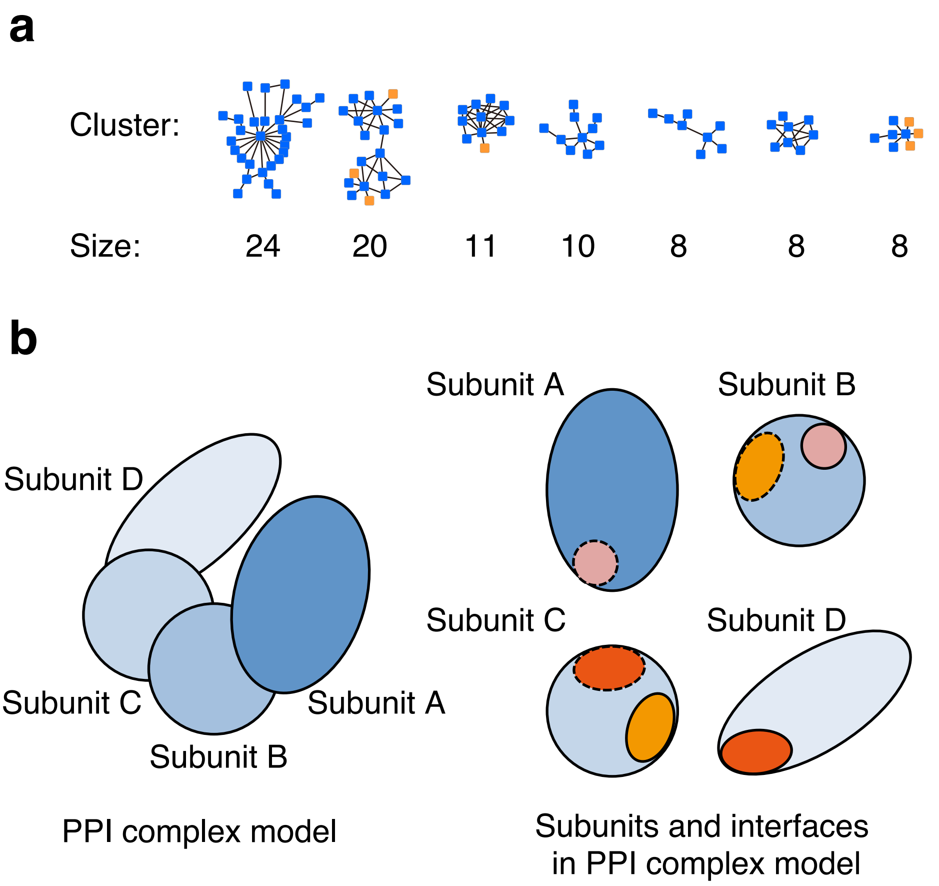


**Figure S2. Schematic explanation for cluster size of sub-network, numbers of subunits and interfaces of PPI complex model**

(a) Examples of clusters (PPI modelable sub-networks) and their cluster sizes. The cluster size is the number of nodes (proteins) in each cluster (isolated sub-graph). (b) An example of PPI complex model and subunit interfaces. Left: blue ellipsoids represent protein subunits in a model. Right: inset ellipsoids represent interfaces, and pairs of interfaces in the same color interact with each other. In this case, the PPI complex model consists of four subunits, and therefore the size of the complex is four. The numbers of interface(s) of subunits A, B, C, and D are 1, 2, 2, and 1, respectively.

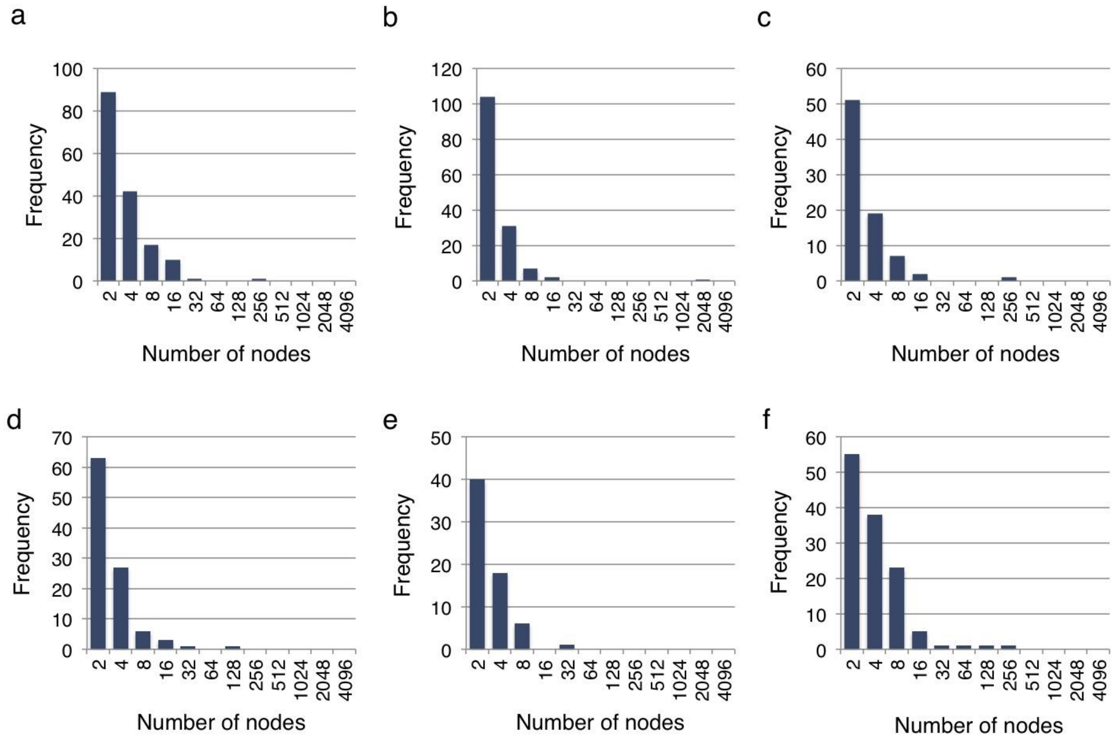


**Figure S3. Modelable PPI sub-networks**

Size distribution of modelable PPI sub-networks of (a) mouse, (b) yeast, (c) bacteria (*Escherichia coli*), (d) insect (*Drosophila melanogaster*), (e) worm (*Caenorhabditis elegans*), and (f) plant (*Arabidopsis thaliana*).

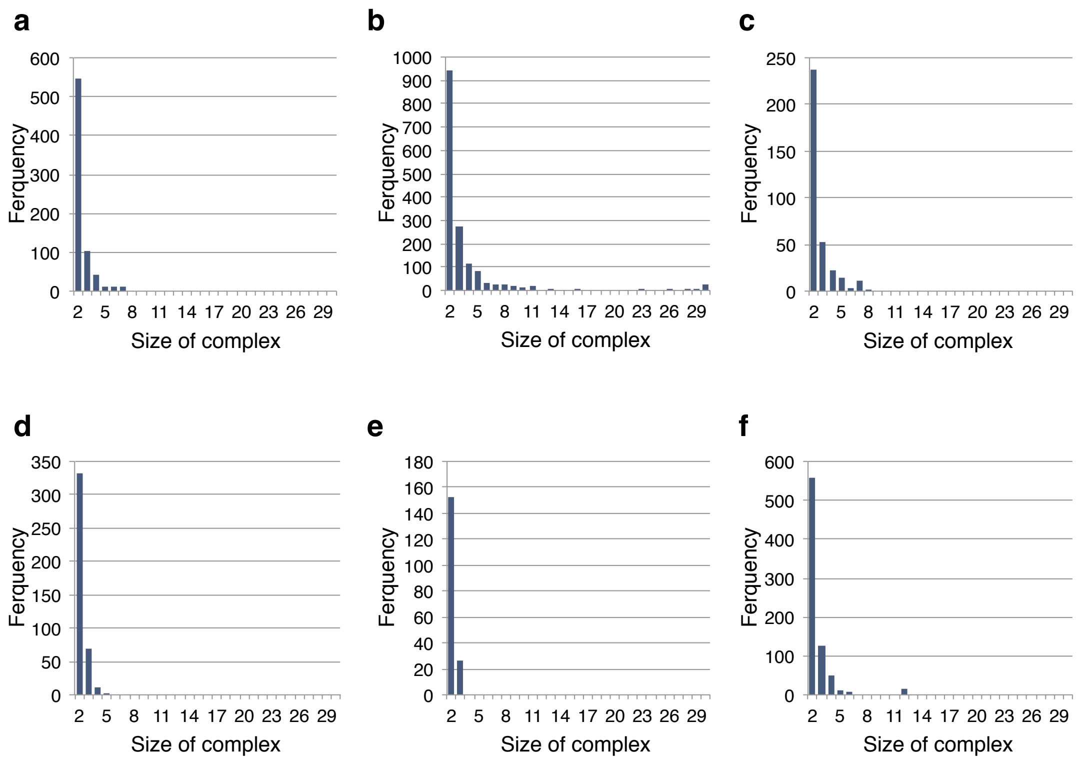


**Figure S4. Size distribution of PPI complex models**

Size distribution of PPI complex models of (a) mouse, (b) yeast, (c) bacteria (*Escherichia coli*), (d) insect (*Drosophila melanogaster*), (e) worm (*Caenorhabditis elegans*), and (f) plant (*Arabidopsis thaliana*).

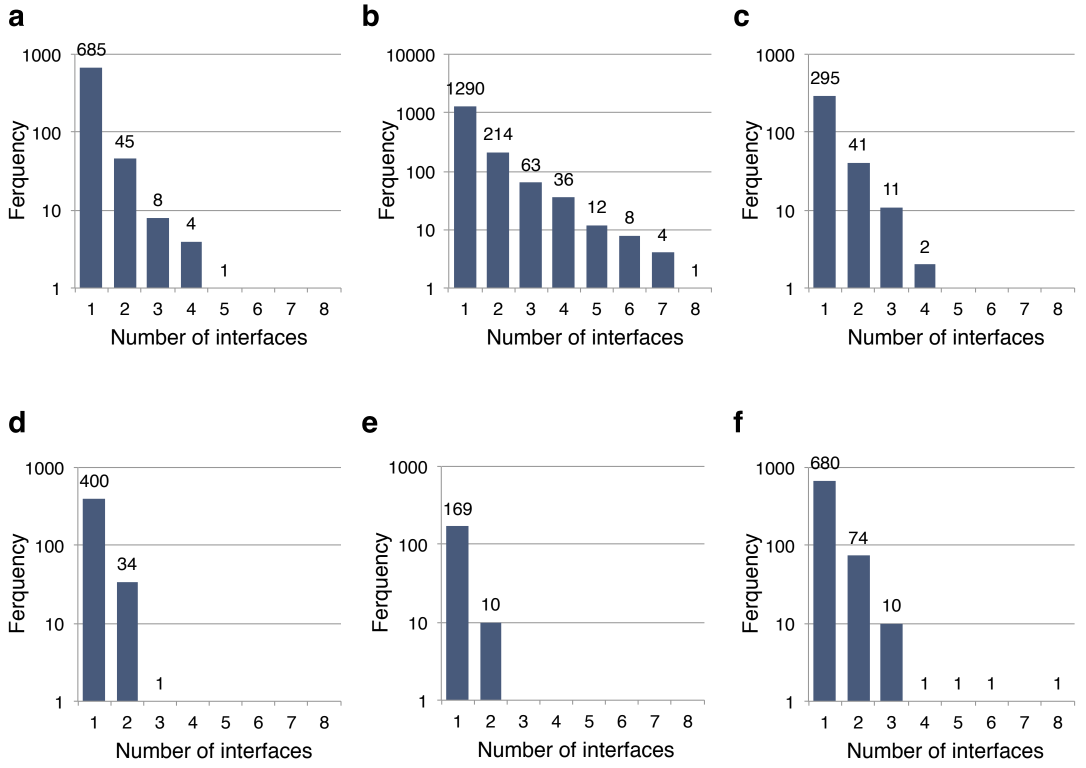


**Figure S5. Interface distribution of PPI complex models**

Number of interface distribution of PPI complex models of (a) mouse, (b) yeast, (c) bacteria (*Escherichia coli*), (d) insect (*Drosophila melanogaster*), (e) worm (*Caenorhabditis elegans*), and (f) plant (*Arabidopsis thaliana*).

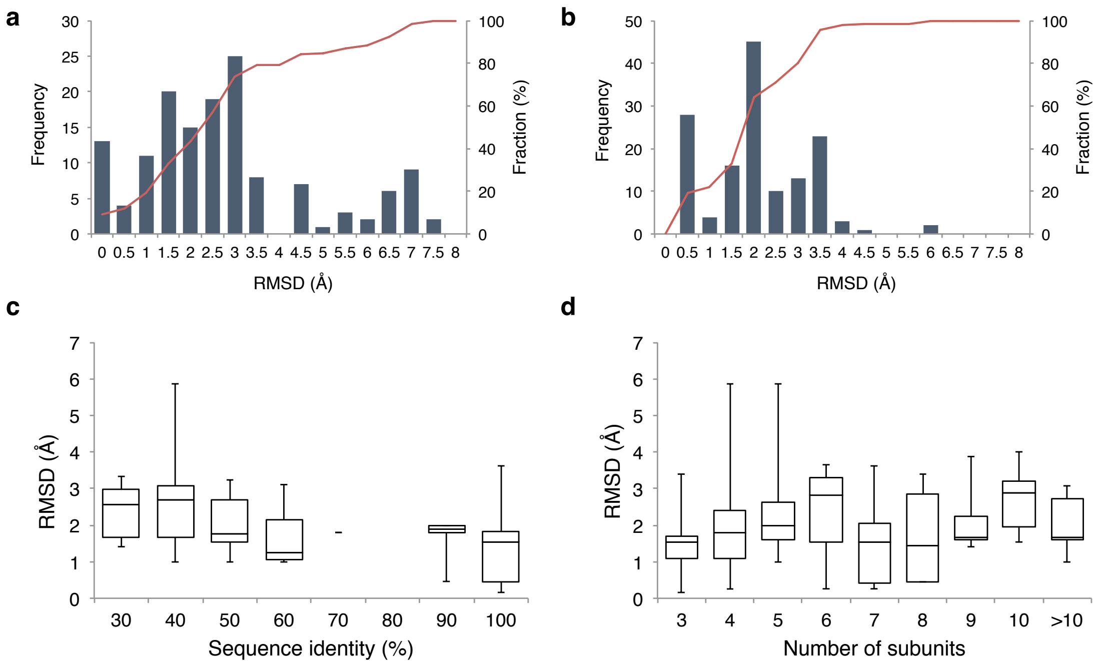


**Figure S6. PPI complex model accuracy**

Distribution of rmsd values for (a) whole complex and (b) predicted subunits in PPI complex models are shown. Bars and lines indicate the frequency (vertical axis and scaled on left) and the cumulative fraction (scaled on right) of models superposed with the corresponding rmsd value (vertical axis) as an upper limit, respectively. The distributions of rmsd (vertical axis) of superposition between template (subunit in corresponding PPI element) and predicted subunits are also shown against (c) amino acid sequence identity between template and target subunits, and (d) number of subunits in corresponding PPI complex model (horizontal axis) in box-and-whisker diagrams.

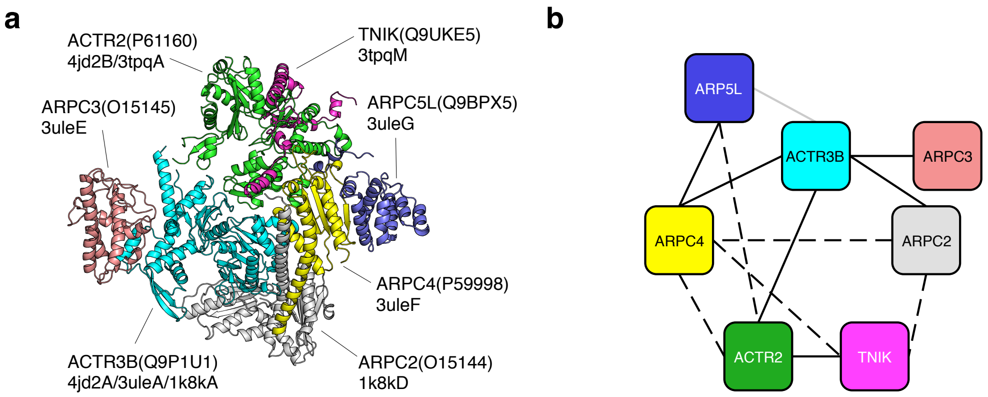


**Figure S7. Indirect interactions in ACTR2 model**

(a) Example of indirect interactions in ACTR2 model. Presented subunits are actin-related protein 2 (ACTR2; green), actin-related protein 3B (ACTR3B; cyan), NCK-interacting protein kinase (TNIK; magenta), actin-related protein 2/3 complex subunit 2 (ARPC2; grey), actin-related protein 2/3 complex subunit 3 (ARPC3; pink), actin-related protein 2/3 complex subunit 4 (ARPC4; yellow) and actin-related protein 2/3 complex subunit 5-like protein (ARPC5L; blue). (b) The modelable PPI sub-network of ACTR2 complex. The solid black, solid grey, and dotted black lines indicate direct, indirect, and model-suggested direct interactions, respectively.

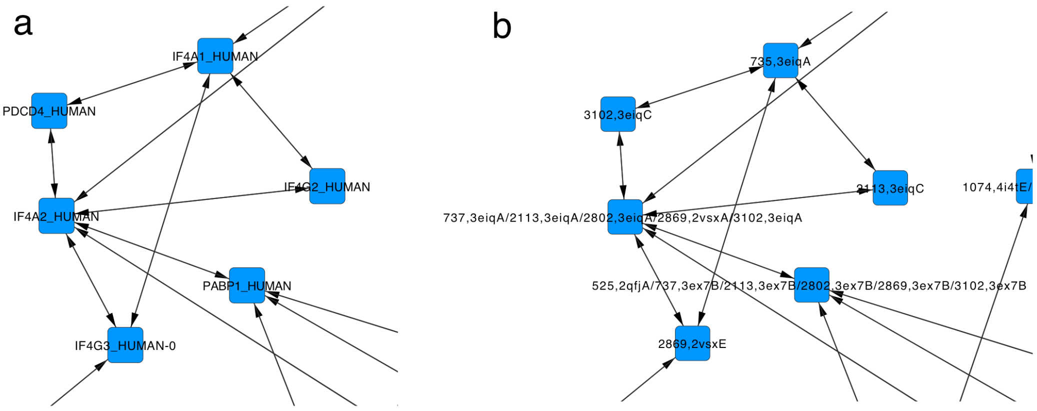


c

| shared name | name | ac | refseq | gene | ▽ model IDs and templates |
|---|---|---|---|---|---|
| IF4G2_HUMAN | IF4G2_HUMAN | P78344 | NP_001166176.1 | EIF4G2 | 2113,3eiqC |
| IF4G3_HUMAN-0 | IF4G3_HUMAN-0 | O43432 | NP_003751.2 | EIF4G3 | 2869,2vsxE |
| PDCD4_HUMAN | PDCD4_HUMAN | Q53EL6 | NP_055271.2 | PDCD4 | 3102,3eiqC |
| PABP1_HUMAN | PABP1_HUMAN | P11940 | XP_005250918.1 | PABPC1 | 525,2qfjA/737,3ex7B/2113,... |
| IF4A1_HUMAN | IF4A1_HUMAN | P60842 | NP_001407.1 | EIF4A1 | 735,3eiqA |
| IF4A2_HUMAN | IF4A2_HUMAN | Q14240 | NP_001958.2 | EIF4A2 | 737,3eiqA/2113,3eiqA/280... |

**Figure S8. PPI elements used for PPI complex modeling**

Modelable PPI sub-networks are summarized in the Supplemental Material (Cytoscape[1] format file ppicomplex_human_network.cys). In this file, each node is annotated with the PDB code and chain ID of PPI elements used in modeling, along with UniProt ID, UniProt accession code, Refseq ID, and gene name. (a) Close-up view of a PPI sub-network labeled with UniProt ID. (b) Same view labeled with PDB codes and chain IDs. (c) The annotations can be also seen with the node table tab of Cytoscape.

**Table S1. Small PPI modelable sub-networks**

| Cluster Size | Uni-Prot AC | No. of membrane proteins | No. of mitochondrial proteins | Annotation |
|---|---|---|---|---|
| 26 | Q9BY76, P05556, P18084, Q9BY67, P16144, P04141, P32927, O75578, Q9UKX5, P56199, P08514, P05106, P17301, P26006, P13612, P26010, P08648, P23229, Q13683, P53708, Q13797, P38570, P06756, P18564, P26012, Q9Y490 | 24 | 0 | Integrin network |
| 20 | P00395, P13073, P09669, Q02221, P00403, P20674, P10606, P15954, P14854, P14406, P47985, P08574, P00156, O14957, P31930, P22695, P07919, P14927, O14949, Q9UDW1 | 20 | 20 | Cytochrome c oxidase and bc1 complex |
| 11 | Q8TF46, Q9NQT5, Q9Y3B2, Q13868, Q9NPD3, Q9NQT4, Q15024, Q96B26, Q5RKV6, Q06265-3, Q9Y2L1 | 0 | 0 | Exosome |
| 10 | Q9Y294, P68431, P62805, Q9NVP2, P49450, Q8NCD3, Q9UER7, P84243, P16104, Q99714 | 0 | 1 | Histone |
| 8 | Q7Z6A9, Q92956, O95971, Q15435, O95407, O43557, Q06643, P01374 | 6 | 0 | TNR |
| 8 | P62324, Q9UIV1, Q9UFF9, P78543, Q96LI5, A5YKK6, Q9ULM6, P50616 | 0 | 0 | CCR4-NOT complex |
| 8 | Q9H845,P36542,P24539, O75947,P18859,P25705, P06576,P30049 | 7 | 8 | ATP synthase |
| 7 | Q9NRF9, Q9NR33, Q14919, Q01658, P25208, Q13952, P23511 | 0 | 0 | DNA polymerase |
| 7 | Q15762, Q92692, P15151, Q15223, Q9NQS3, Q96NY8, Q495A1 | 7 | 0 | Cell adhesion |
| 7 | P05362, P20701, P32942, Q13349, P05107, P11215, P20702 | 7 | 0 | Integrin |
| 7 | O75955, P38606, P21281, Q9Y5K8, O75348, P36543, O95670 | 3 | 0 | ATPase complex |
| 7 | P02511, Q9UJY1, P04792, Q16082, Q12988, O14558, Q9UBY9 | 0 | 0 | Heat shock protein |
| 6 | P02458, P12107, P13942, P20908, P05997, P25940 | 0 | 0 | Collagen |
| 6 | Q9BSD7, O95630, Q15645, Q96FJ0, O94762, Q9UQ90 | 2 | 1 | Signal transduction for cell growth |
| 6 | P57086, Q9P0L1, P49910, O14978, P17028, P17040 | 0 | 0 | Transcription factors |
| 5 | Q15436, O95487, P53992, | 5 | 0 | COPII coat |

| | | | | |
|---|---|---|---|---|
| | Q15437, O94855 | | | |
| 5 | P51858, O75475, Q03164, O00255, Q8N7H5 | 0 | 0 | Transcription |
| 5 | Q13409, P63167, P51808, O96015, Q96FJ2 | 0 | 1 | Dynein |
| 5 | O75396, Q12846, Q13190, Q15836, O75379 | 5 | 0 | Vesicle trafficking |
| 5 | Q8N122, Q9NQL2, Q7L523, Q9HB90, Q5VZM2-2 | 1 | 0 | Ras-related GTP-binding proteins |
| 5 | P78552, P35225, P24394, P05112, P31785 | 3 | 0 | Interleukin |
| 5 | P00492, O60921, Q9NRG1, O60671, Q99638 | 0 | 0 | Cell-cycle checkpoint proteins |
| 5 | P54257, Q8N8D1, O60925, P45378, O00291 | 2 | 1 | Related huntingtin |
| 5 | Q9BXS5, P63010, Q9Y6Q5, O94973, Q96CW1 | 5 | 0 | AP complex |
| 5 | P04439, P30457, P30450, P16188, P30455 | 5 | 0 | HLA1 |
| 5 | P09914, P09913, O14879, Q13325, Q5T764 | 1 | 1 | Interferon induce |
| 4 | Q9UL33, O43617, O75865, Q9Y296 | 0 | 0 | Vesicle transport |
| 4 | Q9Y275, Q02223, O14836, O75888 | 3 | 0 | TNR family |
| 4 | O00470, P31314, P40425, P55347 | 0 | 0 | Homeobox |
| 4 | Q9NZI2, Q9NZV8, Q9NS61-3, Q6PIL6 | 4 | 0 | Potassium channel |
| 4 | P08887, P05231, P40189, P15018 | 2 | 0 | Interleukin-6 |
| 4 | Q05639, P24534, P29692-2, Q01105 | 0 | 0 | Elongation factor |
| 4 | Q9C005, Q8WWB3, P56597, Q96IM9 | 0 | 0 | Acrosome biogenesis |
| 4 | Q01955, P29400, P53420, Q14031 | 4 | 0 | Collagen |
| 4 | Q9H211, O75496, Q14566, D6RGH6 | 0 | 0 | DNA replication |
| 4 | P10747, P33681, P16410, P42081 | 4 | 0 | T-cell activation |
| 4 | P04229, P01903, P13760, P01737 | 3 | 0 | HLA class II |
| 4 | Q06830, Q13162, P32119, P30048 | 0 | 1 | Redox regulation |
| 4 | Q29983, P26718, Q9BZM4, Q6H3X3 | 4 | 0 | Antigen presentation |
| 4 | P25205, O43929, O43913, P33992 | 0 | 0 | DNA replication |
| 4 | P43355, Q8WV22, Q9HAY2, Q96MG7 | 0 | 0 | Chromosome maintenance |
| 4 | P01563, P48551, P05000, P17181 | 2 | 0 | Interferon |
| 4 | P42566, Q8WXE9, O76013, O76009 | 2 | 0 | Growth factor receptor |
| 4 | Q6UY11, Q9BQ66, Q9BYR7, P49901 | 2 | 1 | Regulates adipogenesis |
| 4 | Q8N2Z9, Q96BT3, Q5EE01, A8MT69 | 0 | 0 | Centromere |
| 4 | 78, Q8IW35, 15154, | 0 | 0 | Centrosome |

| | | | | |
|---|---|---|---|---|
| | O43303 | | | |
| 4 | Q99643, P31040, P21912, O14521 | 4 | 4 | Succinate dehydrogenase |
| 3 | P51784, Q13107, O94782 | 0 | 0 | Ubiquitin carboxyl-terminal hydrolase |
| 3 | Q6R6M4, P0C7H9, Q0WX57 | 0 | 0 | Ubiquitin carboxyl-terminal hydrolase |
| 3 | Q9Y5L4, O60220, Q9Y5J9 | 3 | 3 | Mitochondrial translocate |
| 3 | Q99594, Q9GZV5, Q15561 | 1 | 0 | Transcriptional enhancer |
| 3 | Q9Y5J6, P62072, Q9Y5J7 | 3 | 3 | Mitochondrial translocate |
| 3 | P36956-3, P22415, Q15853 | 1 | 0 | Transcription factor |
| 3 | Q53GS9, O75317, P62068 | 0 | 0 | Ubiquitin carboxyl-terminal hydrolase |
| 3 | O15266, P35711, P35712 | 0 | 0 | Transcription factor |
| 3 | Q13950, Q99717, O43541-2 | 0 | 0 | Transcription factor |
| 3 | O15160, Q9Y2S0, P52435 | 0 | 0 | RNA polymerase |
| 3 | Q14995, P51449, Q92753 | 0 | 0 | Nuclear receptor |
| 3 | O75306, O75489, P19404 | 3 | 3 | NADH dehydrogenase |
| 3 | Q92585, P46531, Q06330-3 | 1 | 0 | Transcriptional regulator |
| 3 | O15525, Q16236, O60675 | 0 | 0 | Transcription factor |
| 3 | Q13449, Q9P121, Q7Z3B1 | 3 | 0 | Neurotrimin |
| 3 | P05161, P41226, Q9UMW8 | 0 | 0 | Ubiquitin-like |
| 3 | P29459, P29460, Q9NPF7 | 0 | 0 | Interleukin |
| 3 | P50213, O43837-2, P51553 | 0 | 3 | Dehydrogenase |
| 3 | P20823, Q9H0N5, P61457 | 0 | 0 | Transcriptional regulator |
| 3 | P07359, P00734, P04275 | 1 | 0 | Maintenance of hemostasis |
| 3 | O95390, Q96NZ8, Q8TEU8 | 0 | 0 | Growth differentiation factor |
| 3 | P01225, P23945, P01215 | 1 | 0 | Follicle |
| 3 | P68104, O95257, Q9NQ29-3 | 0 | 0 | DNA replication |
| 3 | O75461, Q14188, Q14186 | 0 | 0 | Transcription factor |
| 3 | P49916, P18887, P06746 | 0 | 0 | DNA repair |
| 3 | P28067, P28068, P02686 | 2 | 0 | HLA class II |
| 3 | Q9NPI6, Q8IU60-2, Q8IZD4 | 0 | 0 | mRNA decapping |
| 3 | P20849, Q14055, Q14050 | 0 | 0 | Collagen |
| 3 | P12109, P12110, P12111 | 1 | 0 | Collagen |
| 3 | P06731, P40199, Q14002 | 3 | 0 | Cell adhesion |
| 3 | P04234, P07766, P09693 | 3 | 0 | T-cell surface glycoprotein |
| 3 | Q53HC0, Q9UPV0, Q86SQ7 | 0 | 0 | Centrosomal |
| 3 | P51587, P60896, Q06609 | 0 | 1 | DNA repair |
| 3 | Q8WZ19, Q13829, Q9H3F6 | 0 | 0 | BTB/POZ domain-containing adapter |
| 3 | P40616, Q8IWJ2, P20340 | 3 | 0 | Membrane traffic |
| 3 | P03950, P13489, P07998 | 1 | 0 | Redox homeostasis |
| 3 | Q9NZD4, P69905, P68871 | 0 | 0 | Hemoglobin |
| 3 | P52594, P51809, O14617 | 2 | 0 | Vesicle transfer |
| 3 | Q9Y4W6, Q8NE63, Q96TA2 | 2 | 2 | Zinc metabolism |
| 3 | P78325, O95633, P19883-2 | 1 | 0 | Related to follicle stimulating hormone |
| 2 | Q8N9L1, Q86UK7 | 0 | 0 | DNA binding protein with zinc finger |
| 2 | P10074, Q96BR9 | 0 | 0 | DNA binding protein with zinc finger |
| 2 | P13010, P12956 | 0 | 0 | Helicase |
| 2 | Q96AX1, Q9H269 | 2 | 0 | Vascular protein |
| 2 | P49767, P35916 | 1 | 0 | Vascular endothelial growth factor receptor |
| 2 | Q9H0E7, Q70CQ1 | 0 | 0 | Ubiquitin carboxyl-terminal hydrolas |
| 2 | Q9UBP6, P57081 | 0 | 0 | tRNA (guanine-N(7)-)-methyltransferase |
| 2 | Q9ULQ1, Q8NHX9 | 2 | 0 | Two-pore calcium channel protein |
| 2 | O15455, Q7L0X0 | 2 | 0 | Toll-like receptor |
| 2 | P28347, P46937-3 | 0 | 0 | Transcriptional enhancer factor |
| 2 | Q9NYB0, Q15554-2 | 0 | 0 | Telomeric repeat-binding factor |
| 2 | P01848, P01850 | 2 | 0 | T-cell receptor |

| | | | | |
|---|---|---|---|---|
| 2 | Q9Y6J9, Q16594 | 0 | 0 | Transcription initiation factor TFIID subunit |
| 2 | Q15544, Q15543 | 0 | 0 | Transcription initiation factor TFIID subunit |
| 2 | P35269, P13984 | 0 | 0 | General transcription factor IIF subunit |
| 2 | Q9UNK0, Q9UEU0 | 2 | 0 | Vesicle transport |
| 2 | P49888, O00204 | 0 | 0 | Sulfotransferase family |
| 2 | Q96H20, Q9BRG1 | 2 | 0 | Vacuolar-sorting |
| 2 | O95470, Q86WV6 | 2 | 1 | Apoptosis |
| 2 | P21673, Q96F10 | 0 | 0 | Diamine acetyltransferase |
| 2 | Q9HCY8, Q96FQ6 | 0 | 0 | Regulation of cell survival and apoptosis |
| 2 | P05109, P06702 | 2 | 0 | Immune response |
| 2 | P63220, P08865 | 1 | 0 | 40S ribosomal protein |
| 2 | O95602, Q9H9Y6 | 0 | 0 | DNA-directed RNA polymerase I subunit |
| 2 | Q9HAT0, Q15506 | 1 | 0 | Sperm |
| 2 | Q13332, P61956 | 1 | 0 | Post-translational modification |
| 2 | Q06323, Q9UL46 | 0 | 0 | Proteasome activator complex subunit |
| 2 | Q9Y248, Q9BRT9 | 0 | 0 | DNA replication complex |
| 2 | A5PKW4, Q96S21 | 2 | 0 | Subunit of Elongin-Cullin-SOCS-box protein |
| 2 | O60486, O75326 | 2 | 0 | Regulating cell migration and immune responses |
| 2 | Q8IXK0-2, Q96GD3 | 0 | 0 | Maintain the transcriptionally repressive state of many genes |
| 2 | O43933, Q13608 | 2 | 0 | Peroxisome biogenesis |
| 2 | P40855, P56589 | 2 | 0 | Peroxisome biogenesis |
| 2 | Q9NZQ7, Q15116 | 2 | 0 | Apoptosis |
| 2 | Q8NC51-4, Q53HC9 | 0 | 0 | Regulation of mRNA stability |
| 2 | P05121, P04004 | 0 | 0 | Major control point in the regulation of fibrinolysis |
| 2 | P68402, Q15102 | 0 | 0 | Inactivates PAF |
| 2 | P08559, P11177 | 0 | 2 | Pyruvate dehydrogenase E1 component subunit |
| 2 | P12694, P21953 | 0 | 2 | 2-oxoisovalerate dehydrogenase |
| 2 | Q7Z3B4, P37198 | 1 | 0 | Component of the nuclear pore complex |
| 2 | P57740, Q8WUM0 | 1 | 0 | Component of the nuclear pore complex |
| 2 | P51843, P35398 | 0 | 0 | Nuclear receptor |
| 2 | P26717, O43914 | 2 | 0 | Recognition of MHCclass I HLA-E |
| 2 | Q9BPW8, O75323 | 0 | 0 | Protein NipSnap homolog |
| 2 | P12829, Q8N2H3 | 0 | 0 | Regulatory light chain of myosin |
| 2 | Q9UKX3, O43586 | 0 | 0 | Regulation of the actin cytoskeleton |
| 2 | Q96EF0, Q96QG7 | 0 | 0 | Myotubularin-related |
| 2 | O15457, O43196 | 0 | 0 | Meiotic recombination |
| 2 | Q13368, Q13636 | 1 | 0 | Membrane trafficking |
| 2 | Q8TD46, P41217 | 2 | 0 | Regulate myeloid cell activity |
| 2 | P08253, P16035 | 0 | 1 | Immune response |
| 2 | Q969V6, Q9ULH7 | 0 | 0 | MKL/myocardin-like protein |
| 2 | Q8N4C8, P10114 | 1 | 0 | Regulation of actin cytoskeleton reorganization |
| 2 | Q9UBB5, Q86YP4 | 0 | 0 | Methyl-CpG-binding |
| 2 | Q9Y2Q5, Q9UHA4 | 2 | 0 | Regulator complex |
| 2 | P00338, P07195 | 0 | 0 | L-lactate dehydrogenase |
| 2 | P11047, O60749 | 2 | 0 | Intracellular trafficking |
| 2 | Q14558, P60891 | 0 | 0 | Synthesis of phosphoribosylpyrophosphate |
| 2 | Q5VWX1, O75525 | 0 | 0 | Regulation of alternative splicing |
| 2 | P54819, O95255 | 2 | 1 | Apoptosis |
| 2 | P57087, Q9BX67 | 2 | 0 | Cell-cell adhesion |

| | | | | |
|---|---|---|---|---|
| 2 | O14896, Q02556 | 0 | 0 | Transcriptional activator |
| 2 | P16871, P13232 | 1 | 0 | Receptor for interleukin-7 |
| 2 | Q01344, P05113 | 1 | 0 | Interleukin-5 and interleukin-5 receptor |
| 2 | Q9Y547, Q9BW83 | 0 | 0 | Component of the IFT complex |
| 2 | P01579, P15260 | 1 | 0 | Interferon gamma and interferon gamma receptor |
| 2 | Q9H496, Q9Y6I4 | 0 | 0 | Torsin-1A-interacting protein |
| 2 | Q13261, P40933 | 1 | 0 | Interleukin-15 and interleukin-15 receptor |
| 2 | Q13651, P22301 | 1 | 0 | Interleukin-10 and interleukin-10 receptor |
| 2 | P14653, P40424-2 | 0 | 0 | Homeobox protein |
| 2 | Q3SXM5, Q00169 | 0 | 1 | Phosphatidylinositol transfer |
| 2 | Q9H2X6, Q9Y6I7 | 0 | 0 | Transcription regulation |
| 2 | Q04756, O43278 | 0 | 0 | HGF activator and Inhibitor of HGF activator |
| 2 | P51610, P14859 | 0 | 0 | Control of the cell cycle |
| 2 | Q96IJ6, Q9Y5P6 | 0 | 0 | Mannose-1-phosphate guanyltransferase |
| 2 | Q8NEA6, Q9UMX1-2 | 0 | 0 | Repressor and activator of transcription |
| 2 | Q8WXD5, Q9H840 | 0 | 0 | Catalyst role in assembly of small nuclear ribonucleoproteins |
| 2 | Q9H3P7, Q08378 | 2 | 1 | Maintaining Golgi structure |
| 2 | Q14397, P35557 | 0 | 0 | Glucokinase regulatory |
| 2 | P30793, P30047 | 1 | 0 | Positively regulates nitric oxide synthesis |
| 2 | Q9H461, P56704 | 1 | 0 | Wnt proteins |
| 2 | O75084, P56703 | 1 | 0 | Wnt proteins |
| 2 | P02794, P02792 | 0 | 0 | Ferritin |
| 2 | P49354, P49356 | 0 | 0 | Protein farnesyltransferase |
| 2 | Q9BXW9-2, Q9NVI1 | 0 | 0 | Fanconi anemia group |
| 2 | P08709, P13726 | 1 | 0 | Blood coagulation |
| 2 | Q92731, A7E2Y1 | 1 | 0 | Muscle contraction |
| 2 | P62495, Q8IYD1 | 0 | 0 | Translation termination in response to termination codons |
| 2 | P19235, P01588 | 1 | 0 | Erythropoietin |
| 2 | Q8TC92, Q16206 | 2 | 0 | Ecto-NOX disulfide-thiol exchanger |
| 2 | Q96AY2, Q96NY9 | 0 | 0 | Crossover junction endonuclease |
| 2 | Q13347, Q9Y4P8 | 1 | 0 | Eukaryotic translation initiation factor 3 (eIF-3) complex |
| 2 | Q9UNE0, Q92838 | 2 | 0 | Mediates the activation of NF-kappa-B and JNK |
| 2 | O77932, Q9H0D6 | 0 | 0 | Exoribonuclease activity |
| 2 | P01909, P01920 | 2 | 0 | HLA class II histocompatibility antigen |
| 2 | P54098, Q9UHN1 | 0 | 2 | DNA polymerase subunit |
| 2 | P49005, Q15054 | 0 | 0 | DNA polymerase delta |
| 2 | Q14181, P09884 | 0 | 0 | DNA polymerase alpha |
| 2 | Q9UKG1, Q8NEU8 | 2 | 0 | DCC-interacting protein |
| 2 | P49917, Q13426 | 0 | 0 | DNA repair |
| 2 | P09622, O00330 | 0 | 2 | Pyruvate dehydrogenase protein X component |
| 2 | P26196, Q96F86 | 0 | 0 | mRNA decapping |
| 2 | P43146, O95631 | 1 | 0 | Netrin |
| 2 | P78310, Q86YT9 | 2 | 0 | Junctional adhesion |
| 2 | O60494, P27352 | 1 | 0 | Cubilin |
| 2 | O14936, O95395 | 2 | 0 | Peripheral plasma membrane protein CASK |
| 2 | P34998, P06850 | 1 | 0 | Corticoliberin |
| 2 | Q9UGL9, Q96ID5 | 0 | 0 | Immunoglobulin superfamily member |
| 2 | P78560, Q9HB75 | 0 | 0 | Apoptosis |

| | | | | |
|---|---|---|---|---|
| 2 | Q5TA50, Q86YI8 | 1 | 0 | Modulates chromatin structure |
| 2 | P53621, O14579 | 1 | 0 | Coatomer subunit |
| 2 | P27658, P25067 | 2 | 0 | Collagen alpha-1(VIII) |
| 2 | P02462, P08572 | 2 | 0 | Collagen alpha-1(IV) |
| 2 | P02452, P08123 | 0 | 0 | Collagen alpha-1(I) |
| 2 | O75175, Q9HCJ0 | 0 | 0 | RNA-mediated gene silencing |
| 2 | P61604, P10809 | 0 | 2 | Heat shock protein |
| 2 | Q9HCU4, Q8WUB8 | 1 | 0 | Cell/cell signaling in nervous system |
| 2 | Q5SZQ8, A7XYQ1 | 0 | 0 | Regulation of pre-mRNA alternative splicing |
| 2 | Q08722, Q9P1W8 | 2 | 0 | Cell adhesion |
| 2 | P06729, P19256 | 2 | 0 | Mediate adhesion between T-cells and other cell types |
| 2 | Q9BZW8, P09326 | 2 | 0 | Modulate other receptor-ligand interactions |
| 2 | Q5M9N0, O94868 | 0 | 0 | Coiled-coil |
| 2 | Q8NEF3, Q8N6V9 | 0 | 0 | Coiled-coil |
| 2 | P63098, Q08209 | 2 | 0 | Regulatory subunit of calcineurin |
| 2 | Q16602, O60894 | 2 | 0 | Receptor for calcitonin-gene-related peptide |
| 2 | Q9P0X4, Q96P56 | 2 | 0 | Voltage-sensitive calcium channels |
| 2 | O60238, Q12983 | 2 | 2 | Apoptosis |
| 2 | P18075, Q13253 | 0 | 0 | Induces cartilage and bone formation |
| 2 | P12643, P36894 | 1 | 0 | Induces cartilage and bone formation |
| 2 | P54132, Q14191 | 0 | 0 | DNA replication |
| 2 | O00499, Q9UBW5 | 1 | 0 | Regulation of synaptic vesicle endocytosis |
| 2 | Q9BUH8, Q9H4E7 | 2 | 0 | Sustain the structure of postsynaptic density |
| 2 | P23560, P20783 | 0 | 0 | Brain-derived neurotrophic factor |
| 2 | O15265, Q9Y4A5 | 0 | 0 | Mediates the interaction of STAGA complex |
| 2 | O43488, O95154 | 0 | 0 | Aflatoxin B1 aldehyde reductase member |
| 2 | Q8NFD5, P51531 | 0 | 0 | Transcriptional activation and repression |
| 2 | Q8N6T3-2, Q5U651 | 0 | 0 | Involved in membrane trafficking and /or vesicle transport |
| 2 | O14744, Q9BQA1 | 0 | 0 | Arginine methyltransferase |
| 2 | P04075, P05062 | 0 | 0 | Glycolysis and gluconeogenesis |
| 2 | P00352, P05091 | 0 | 1 | Retinal dehydrogenase |
| 2 | Q13155, Q15046 | 0 | 1 | Mediates ubiquitination and degradation of FUBP1 |
| 2 | Q9H9G7, Q5T9A4 | 1 | 1 | Mitochondrial network organization typical for stem cells |
| 2 | P05141, P12236 | 2 | 2 | ADP/ATP translocase |
| 2 | P28288, Q9BRX2 | 1 | 0 | Recognizing stalled ribosomes and triggering |
| 2 | P01009, P08246 | 0 | 0 | Modifies functions of natural killer cells |

## Table S2. Disease-related variants mapped on model-suggested interfaces

| Disease-causing protein coding gene*1 | Variant | Disease name | OMIM ID | Interacting protein coding gene |
|---|---|---|---|---|
| Variants on suggested interface | | | | |
| PTPN11 (Q06124) | A465T | LEOPARD syndrome 1 | 151100 | FN1 (P02751) |
| BUB1B (O60566) | R36Q | Premature chromatid separation trait | 176430 | MAD2L1 (Q13257) |
| GNB4 (Q9HAV0) | G53D<br>K89E | Charcot-Marie-Tooth disease, dominant, intermediate type, F | 615185 | GNAI3 (P08754) |
| TGFBR1 (P36897) | P83L | Multiple self-healing squamous epithelioma | 132800 | TGFBR2 (P37173) |
| PEX10 (O60683) | H290Q | Peroxisome biogenesis disorder 6B | 614871 | SUMO3 (P55854) |
| TUBA1A (Q71U36) | R402H, R402C<br>S419L | Lissencephaly 3 | 611603 | TNIP2 (Q8NFZ5)<br>TNIP2 (Q8NFZ5) |
| MKRN3 (Q13064) | R365S | Precocious puberty, central 2 | 615346 | SUMO3 (P55854) |
| PFN1 (P07737) | M114T<br>G118V | Amyotrophic lateral sclerosis 18 | 614808 | DSTN (P60981) |
| WAS (P42768) | A236E | Thrombocytopenia 1 | 313900 | CDC42BPA (Q5VT25) |
| TUBB4A (P04350) | D249N | Leukodystrophy, hypomyelinating, 6 | 612438 | TUBA1A (Q71U36) |
| Variants on suggested and experimentally detected interface | | | | |
| VHL (P40337) | K159E<br>E186K | Von Hippel-Lindau disease | 193300 | CUL3 (Q13618) |
| TCAP (O15273) | R87Q | Cardiomyopathy, dilated 1N | 607487 | OBSCN (Q5VST9) |
| FAS (P25445) | G253D, G253S | Autoimmune lymphoproliferative syndrome 1A | 601859 | TNFRSF1A (P19438) |
| BTK (Q06187) | G613D<br>C633Y<br>R641C | X-linked agammaglobulinemia | 300755 | CSRP1 (P21291) |
| CDKN2A (P42771) | R107C<br>L117M<br>A118T<br>G122R | Melanoma, cutaneous malignant 2 | 155601 | SRPK1 (Q96SB4) |
| TP53 (P04637) | R175G, R175L, R175H<br>R174G<br>E180K | Li-Fraumeni syndrome | 151623 | PPP1CA (P62136)<br>PPP1CA (P62136)<br>PPP1CA (P62136) |
| CHEK2 (O96017) | G167R<br>R145P<br>R180C, R180H | Prostate cancer | 176807 | PDGFRA (P16234)<br>ANKRD44 (Q8N8A2)<br>EPHA3 (P29320) |
| OPTN (Q96CV9) | E478G<br>A288G | Amyotrophic lateral sclerosis 12 | 613435 | RABGEF1 (Q9UJ41)<br>NUDC (Q9Y266) |
| IKBKG (Q9Y6K9) | D311N | Ectodermal dysplasia, anhidrotic, with immunodeficiency X-linked | 300291 | PAK2 (Q13177),<br>NFKB1 (P19838),<br>RIPK3 (Q9Y572),<br>RABGEF1 (Q9UJ41) |
| PIK3R1 (P27986) | R649W | SHORT syndrome | 269880 | NCK1 (P16333),<br>PTPN11 (Q06124),<br>PLCG1 (P19174) |
| CDK4 (P11802) | N41S | Melanoma, cutaneous malignant 3 | 609048 | CCNB1 (P14635) |
| SDHD (O14521) | Y114C | Paragangliomas 1 | 168000 | SDHB (P21912) |
| WAS (P42768) | L270P | Neutropenia, severe congenital, X-linked | 300299 | DDX46 (Q7L014),<br>PDE6D (O43924) |
| | A323P | Incontinentia pigmenti | 308300 | PAK2 (Q13177) |
| IKBKG (Q9Y6K9) | E315A<br>R319Q | X-linked familial atypical mycobacteriosis 1 | 300636 | PAK2 (Q13177),<br>PIK3R1 (P27986)<br>PAK2 (Q13177),<br>RABGEF1 (Q9UJ41) |
| NCF2 (P19878) | R184P | Granuloma | 233710 | PAK2 (Q13177),<br>MYH9 (P35579) |

*1 UniProt accession codes are in parentheses.

**Table S3. PPI complex model-suggested indirect interactions and experimental method**

| Method | No. of total interactions | No. of indirect interactions | Rate (%) |
|---|---|---|---|
| Co-immunoprecipitation | 2804 | 592 | 21.1 |
| Two hybrid | 3349 | 438 | 13.1 |
| Tandem affinity purification | 968 | 192 | 19.8 |
| Pull down | 487 | 69 | 14.2 |
| Co-sedimentation | 106 | 23 | 21.7 |
| Peptide array | 237 | 22 | 9.3 |
| Comigration in non-denaturing gel electrophoresis | 26 | 16 | 61.5 |
| Protein kinase assay | 150 | 15 | 10.0 |
| Blue native page | 45 | 15 | 33.3 |
| Molecular sieving | 90 | 13 | 14.4 |
| Enzymatic study | 80 | 11 | 13.8 |
| X-ray crystallography | 194 | 10 | 5.2 |
| Fluorescence microscopy | 57 | 8 | 14.0 |
| Confocal microscopy | 57 | 7 | 12.3 |
| Chromatography | 71 | 6 | 8.5 |
| Protein array | 77 | 5 | 6.5 |
| Proximity ligation assay | 70 | 5 | 7.1 |
| Enzyme linked immunosorbent assay | 30 | 4 | 13.3 |
| Isothermal titration calorimetry | 30 | 4 | 13.3 |
| Display technology | 20 | 4 | 20.0 |
| Cross-linking study | 48 | 3 | 6.3 |
| Nuclear magnetic resonance | 18 | 3 | 16.7 |
| Biochemical | 6 | 2 | 33.3 |
| Protease assay | 6 | 2 | 33.3 |
| Array technology | 4 | 2 | 50.0 |
| Imaging technique | 4 | 2 | 50.0 |
| Deacetylase assay | 2 | 2 | 100.0 |
| Ion exchange chromatography | 2 | 2 | 100.0 |
| Proximity-dependent biotin identification | 23 | 1 | 4.3 |
| Bimolecular fluorescence complementation | 14 | 1 | 7.1 |
| Antibody array | 8 | 1 | 12.5 |
| Phosphatase assay | 180 | 0 | 0.0 |
| Surface plasmon resonance | 62 | 0 | 0.0 |
| Fluorescence polarization spectroscopy | 31 | 0 | 0.0 |
| Far Western blotting | 26 | 0 | 0.0 |
| Fluorescent resonance energy transfer | 24 | 0 | 0.0 |
| Luminescence-based mammalian interactome mapping | 20 | 0 | 0.0 |
| Fluorescence-activated cell sorting | 14 | 0 | 0.0 |
| Ubiquitin reconstruction | 14 | 0 | 0.0 |
| Affinity technology | 12 | 0 | 0.0 |
| Competition binding | 8 | 0 | 0.0 |
| Mammalian protein-protein interaction trap | 8 | 0 | 0.0 |
| Comigration in SDS PAGE | 6 | 0 | 0.0 |
| Electron microscopy | 6 | 0 | 0.0 |
| Solid phase assay | 6 | 0 | 0.0 |
| GDP/GTP exchange assay | 4 | 0 | 0.0 |
| Genetic interference | 4 | 0 | 0.0 |
| Reverse phase chromatography | 4 | 0 | 0.0 |
| Saturation binding | 4 | 0 | 0.0 |
| Scintillation proximity assay | 4 | 0 | 0.0 |
| Amplified luminescent proximity homogeneous assay | 2 | 0 | 0.0 |
| Atomic force microscopy | 2 | 0 | 0.0 |
| Beta galactosidase complementation | 2 | 0 | 0.0 |
| Electrophoretic mobility-based method | 2 | 0 | 0.0 |
| Enzymatic footprinting | 2 | 0 | 0.0 |

| | | | |
|---|---|---|---|
| Filter binding | 2 | 0 | 0.0 |
| Gal4 vp16 complementation | 2 | 0 | 0.0 |
| GTPase assay | 2 | 0 | 0.0 |
| Lex-a dimerization assay | 2 | 0 | 0.0 |
| Protease accessibility laddering | 2 | 0 | 0.0 |
| Protein three hybrid | 2 | 0 | 0.0 |
| X-ray scattering | 2 | 0 | 0.0 |

## Reference

1. Shannon P., Markiel A., Ozier O., Baliga N. S., Wang J. T., Ramage D., Amin N., Schwikowski B. & Ideker T., Cytoscape: a software environment for integrated models of biomolecular interaction networks. *Genome Res.*, **13,** 2498-2504 (2003)
